# Lipid-Based Drug Delivery Systems as Emerging Tools to Overcome Antifungal Resistance

**DOI:** 10.3390/ijms27104487

**Published:** 2026-05-16

**Authors:** Lide Arana, Andrea Guridi, Elena Sevillano, Esther Tamayo, Elena Eraso, Itziar Alkorta, Ianire Mate

**Affiliations:** 1 Department of Applied Chemistry, University of the Basque Country EHU, 20018 Donostia, Spain; lide.arana@ehu.eus; 2 Department of Immunology, Microbiology and Parasitology, Faculty of Medicine and Nursing, University of the Basque Country EHU, 48940 Leioa, Spain; andrea.guridi@ehu.eus (A.G.); elena.sevillano@ehu.eus (E.S.); esther.tamayo@ehu.eus (E.T.); elena.eraso@ehu.eus (E.E.); 3 Department of Biochemistry and Molecular Biology, Faculty of Science and Technology, University of the Basque Country EHU, 48940 Leioa, Spain; itzi.alkorta@ehu.eus

**Keywords:** lipid-based drug delivery systems, antifungal therapy, liposomes (LPs), solid lipid nanoparticles (SLNs), nanostructured lipid carriers (NLCs), lipid nanoparticles (LNPs), antifungal resistance, efflux pump inhibition, biofilms, dual-drug delivery

## Abstract

Fungal infections represent an escalating global health challenge due to their increasing incidence, the emergence of multidrug-resistant pathogens, and the limited development of new antifungal agents. Therapeutic efficacy is compromised by mutations in drug targets, overexpression of efflux pumps, alterations in the ergosterol biosynthetic pathway, biofilm-associated tolerance, and extensive genomic plasticity. The growing prevalence of antifungal resistance and the limited availability of effective therapeutic options highlight the urgent need to strengthen epidemiological surveillance and accelerate research into innovative therapeutic strategies. In this review, we discuss the potential of lipid-based drug delivery systems (LDDSs) as a versatile strategy to optimize antifungal administration and overcome resistance mechanisms. Liposomes (LPs), solid lipid nanoparticles (SLNs), nanostructured lipid carriers (NLCs), and lipid nanoparticles (LNPs) offer high biocompatibility, efficient encapsulation of hydrophobic compounds, structural stability, and controlled drug release. Their nanoscale properties facilitate penetration into biofilms, promote intracellular uptake, and reduce the impact of efflux-mediated drug extrusion, thereby improving cellular penetration and circumventing resistance pathways. In addition, LDDSs increase bioavailability, reduce toxicity, and promote drug accumulation within poorly accessible tissue compartments. Overall, LDDSs represent a promising approach to expand the therapeutic arsenal against both superficial and invasive fungal infections, particularly those caused by multidrug-resistant pathogens.

## 1. Introduction

Fungal infections encompass a spectrum of conditions ranging from superficial infections, which are generally easier to manage, to invasive infections, which are more severe, challenging to treat, and associated with high mortality rates (exceeding 50%) [1]. Superficial and mucocutaneous mycoses, although rarely life-threatening, remain extremely common worldwide and contribute substantially to chronic morbidity and recurrent antifungal use. Despite their impact, they have historically received limited attention and resources; however, emerging epidemiological data underscore the critical burden and severity of invasive fungal disease, which constitute a major public health concern [2]. Furthermore, the growing prevalence of antifungal resistance and the limited availability of effective therapeutic options highlight the urgent need for enhanced surveillance and research efforts [3].

Current global estimates indicate an annual incidence of approximately 6.5 million invasive fungal infections, resulting in about 3.8 million deaths, of which approximately 2.5 million (≈68%) are directly attributable to the infections [1]. Moreover, these infections exacerbate morbidity and mortality in comorbid conditions. Predisposing factors include immunosuppressive states, such as hematological or solid-organ cancers, transplantation, HIV infection, prolonged corticosteroid or immunomodulatory therapy, and extended ICU stays [4,5]. Notably, during the COVID-19 pandemic, secondary fungal infections increased markedly, with an incidence ranging from 5% to 26.7% [6,7]. The predominant genera responsible for invasive fungal infections are *Aspergillus*, *Candida*, and related genera including *Candidozyma*, *Meyerozyma*, *Nakaseomyces*, *Pichia*, and *Cryptococcus*, with *Pneumocystis* also contributing substantially [1,7]. A recent systematic assessment estimated that invasive aspergillosis accounts for ~2.11 million cases and ~1.80 million deaths annually (85% mortality), whereas candidemia affects ~1.57 million individuals with ~995,000 deaths (63.6%). Cryptococcal meningitis causes ~194,000 cases and 147,000 deaths (75.8%), while pneumocystis pneumonia impacts ~505,000 people, resulting in ~214,000 fatalities (42.4%) each year [1].

To treat fungal infections, five major classes of antifungal agents are currently available: azoles (imidazoles: miconazole, ketoconazole; triazoles: fluconazole, voriconazole, etc.; and tetrazoles: oteseconazole), echinocandins (caspofungin, micafungin, anidulafungin, and rezafungin), polyenes (amphotericin B, nystatin, and natamycin), antimetabolites (flucytosine), and allylamines (terbinafine). Despite this diversity, there is an increasing number of resistant isolates, while novel drug development remains limited. Over the past decade, only four new antifungals have been approved: oteseconazole (2022) and ibrexafungerp (2021), both for superficial infections; isavuconazole (2015), for invasive aspergillosis and mucormycosis; and rezafungin (2023), for adult candidemia and invasive candidiasis [8]. Despite the critical need for new therapies, there are only nine agents in development, as shown in the report carried out by the World Health Organization (WHO) [3]. In addition, the available antifungals have significant pharmacological limitations, such as low solubility, dose-dependent toxicity, poor tissue penetration, drug interactions, and reduced efficacy against biofilms. These limitations compromise clinical efficacy and promote the development of resistance, especially in invasive infections and immunocompromised patients.

The increasing reports of antifungal resistance, combined with the limited development of new therapeutic molecules, prompted the WHO to publish, in 2022, the first fungal priority pathogens list, emphasizing the urgent need for novel treatment strategies [9]. This list includes 19 species categorized into three priority tiers. Critically, the highest priority group comprises four major fungal pathogens. *Cryptococcus neoformans* causes life-threatening cryptococcosis, particularly in HIV patients, with mortality up to 61% despite therapy. *Candidozyma auris* (formerly *Candida auris*) is an emerging multidrug-resistant yeast linked to hospital outbreaks, showing high fluconazole resistance (87–100%) and mortality of 29–53%. *Aspergillus fumigatus*, an environmental mold, is responsible for invasive aspergillosis; azole resistance is rising globally, with mortality reaching 88% or higher. *Candida albicans* remains a leading cause of invasive candidiasis worldwide, with a mortality of 20–50%, though its incidence is declining relative to other species [9,10].

Although intrinsic antimicrobial resistance is a natural phenomenon, the rising resistance rates are partly driven by prolonged treatment regimens and the widespread use of antifungal agents in immunocompromised patients [11]. Furthermore, the agricultural application of certain antifungals, such as azoles, has been directly linked to an increase in resistant isolates, notably in *Aspergillus* species [12]. Resistance may be intrinsic—due to reduced drug-binding affinity, efflux pump activity, or alterations in cell wall composition—or acquired through genetic changes such as point mutations, gene duplication, transposon insertions, aneuploidy, and loss of heterozygosity. Additional factors include phenotypic heterogeneity, such as biofilm formation and stress-response activation, and epigenetic mechanisms that regulate gene expression without altering the underlying DNA sequence, enabling rapid fungal adaptation to antifungal agents [13,14]. Antifungal resistance results in therapeutic failure, prolonged infections, increased healthcare costs, and higher morbidity and mortality.

In this context, nanoparticle-based technologies have emerged as promising tools for optimizing the administration of antifungal agents. Lipid-based drug delivery systems (LDDSs), including liposomes (LPs), solid lipid nanoparticles (SLNs), nanostructured lipid carriers (NLCs) and lipid nanoparticles (LNPs), stand out for their biocompatibility, ability to incorporate hydrophobic compounds, physicochemical stability, and potential to modulate drug release. These characteristics make them ideal delivery systems for improving bioavailability, reducing toxicity, and overcoming resistance mechanisms such as biofilm formation or efflux pump overexpression. It is essential to highlight that these nanoparticles are not limited to invasive fungal infections. They are also widely used to enhance the topical administration of antifungals in superficial infections, improving penetration into the skin and mucous membranes, local retention and therapeutic efficacy.

This article is presented as a narrative review aimed at providing a comprehensive and integrative overview of LDDSs in the context of antifungal resistance. The literature was selected based on its relevance to antifungal resistance mechanisms, lipid-based formulation strategies, and translational applicability, with particular emphasis on peer-reviewed experimental, preclinical, and clinical studies. Given the heterogeneity of the reported outcomes—ranging from in vitro parameters such as MIC reduction, biofilm inhibition, and intracellular drug accumulation to pharmacokinetic and in vivo efficacy data—findings were interpreted qualitatively rather than through direct quantitative comparison. In this context, results were analyzed considering the experimental model, fungal species, antifungal agent, formulation type, and study design, with greater weight given to studies providing mechanistic insights or in vivo validation. This approach allowed us to integrate diverse types of evidence while preserving their specific experimental context.

Taken together, the high clinical burden of fungal infections, the rising prevalence of antifungal resistance, and the limited development of new antifungal agents underscore an urgent therapeutic gap. In this context, the central premise of this review is that LDDSs have the potential to expand current antifungal therapeutic options by overcoming key pharmacokinetic limitations and resistance-related barriers, thereby restoring or enhancing the clinical utility of existing antifungal drugs.

## 2. Antifungal Drugs: Mechanisms, Spectrum, and Emerging Resistance

### 2.1. Azoles

Azoles represent a commonly used class of antifungal drugs, which are categorized into triazoles (fluconazole, isavuconazole, itraconazole, posaconazole and voriconazole), imidazoles (clotrimazole, miconazole, econazole, ketoconazole, luliconazole, sulconazole, etc.) and tetrazoles (oteseconazole).

They inhibit the synthesis of ergosterol, an essential component of the fungal cell membrane. Their target is the fungal cytochrome P450-dependent lanosterol 14α-demethylase enzyme (encoded by the *ERG11* gene in yeasts and *CYP51* in filamentous fungi), which catalyzes the conversion of lanosterol to ergosterol. This results in ergosterol depletion that increases membrane permeability and the accumulation of toxic sterol intermediates produced by the Δ-5,6-desaturase (*ERG3*) [15,16,17,18].

Broad-spectrum triazoles, which may exhibit fungistatic or fungicidal effects depending on the organism and drug concentration, are primarily used for the treatment of systemic and invasive fungal infections, including candidiasis and aspergillosis. In contrast, imidazoles are more commonly used topically or for mucocutaneous infections, whereas oteseconazole is primarily administered orally and has been specifically developed for the treatment of recurrent vulvovaginal candidiasis [19,20,21,22].

Nevertheless, azoles can cause hepatotoxicity, particularly ketoconazole and voriconazole. Therefore, regular monitoring of liver enzymes is recommended during prolonged use. Long-term treatment has also been associated with neurotoxic or sensory effects such as peripheral neuropathy, visual disturbances and photosensitivity, especially with voriconazole and itraconazole [23,24].

Furthermore, azole resistance among *Candida* and *Aspergillus* species has been identified as a significant challenge for treatment success [10,25]. Therefore, the selection of specific azoles and dosing should be guided by species identification, susceptibility testing, and site of infection [21,22]. Several mechanisms of fungal resistance to azoles have been described, such as (i) overexpression of efflux pumps, (ii) mutations or overexpression of the azole target enzyme, and (iii) alterations in the ergosterol biosynthesis pathway or genomic plasticity.

(i) The upregulation of efflux pumps, which reduce intracellular drug accumulation, has been often associated with resistance to azoles. In the case of *C. albicans*, upregulation of the ATP-binding cassette transporters (ABC-Ts), Cdr1 and Cdr2, and major facilitator transporter Mdr1 have been described as important mechanisms. This process is regulated by mutations in transcriptional activator genes *TAC1* and *MRR1*, respectively [26,27,28]. Similarly, in *C. auris*, gain of function mutations in homolog genes *TAC1B* and *MRR1A* confer resistance to fluconazole [29,30,31]. In *A. fumigatus*, overexpression of ABC-Ts such as AtrF also mediates azole resistance [32].
(ii) With regard to alterations in drug targets, mutations in genes *ERG11* in yeast and *CYP51* in filamentous fungi have been shown to decrease azole-binding affinity. In *C. albicans* more than 140 amino acid substitutions have been reported in the *ERG11* gene, most of them clustered in three hotspot regions: 105–165, 266–287 and 405–488 [33,34,35]. Resistance-conferring mutations in *C. auris* have also been reported [36]. In filamentous fungi, particularly in *A. fumigatus*, resistance is attributable frequently to point mutations, mainly in the *CYP51A* gene. Mutations such as TR34/L98H and TR46/Y121F/T289A have been associated with previous environmental or patient azole exposure [37,38].
  Increased drug target expression of *ERG11*, often due to mutations in the zinc cluster transcription factor gene *UPC2*, also confers resistance in *C. albicans* and *C. auris* [39,40]. In *A. fumigatus* overexpression is regulated by the sterol regulatory element-binding protein (SREBP) and the fungal-specific transcription factors AtrR and SltA [41,42].
(iii) Additional resistance mechanisms include alterations in other ergosterol pathway genes, such as mutations in *ERG3* that mitigate the toxicity of accumulated intermediates [43], as well as genomic alterations, including aneuploidy that increases the copy number of resistance-associated genes, among others. In the case of *C. albicans*, the duplication of the left arm of chromosome 5 results in the formation of an isochromosome(i(5L))which in turn leads to an increase in the *ERG11* gene and the transcription factor regulating efflux pump *TAC1* [44]. Biofilm formation, which impedes antifungal penetration, contributes substantially to broad antifungal resistance not only by reducing drug permeability but also through the presence of persistent cells and specialized extracellular matrix components that further enhance tolerance to antifungal agents.

### 2.2. Echinocandins

Echinocandins represent a major class of antifungal agents widely used in the treatment of invasive candidiasis and aspergillosis [45,46]. This class of antifungal drugs comprises fungal secondary metabolites characterized by a cyclic lipopeptide core and lipid residues that determine their antifungal activity. The most representative compounds of this group are caspofungin, micafungin and anidulafungin, as well as the more recently developed rezafungin, which exhibits a prolonged half-life and potential for weekly administration. Their mechanism of action is based on a non-competitive and concentration-dependent inhibition of 1,3-β-D-glucan synthase, an essential enzyme for β-glucan synthesis, a fundamental structural component of the fungal cell wall. Specifically, echinocandins target the catalytic subunits of glucan synthase, encoded by the *FKS1* and *FKS2* genes, causing disruption of glucan synthesis, osmotic instability, cell lysis and death in most susceptible species [46,47]. This mechanism underlies their high selectivity, low toxicity to human cells, and minimal drug interaction potential.

Regarding their spectrum of activity, echinocandins are fungicidal against most *Candida* species and fungistatic against *Aspergillus* [48], which limits their use in aspergillosis to cases refractory to or intolerant of azoles [49]. Their activity does not extend to *Cryptococcus* or *Mucorales*, reflecting a narrower spectrum compared to polyenes.

Currently, their clinical use is mainly restricted to hospitals, where they are administered intravenously in cases of severe infection such as invasive candidiasis, with approximately 60% of patients with candidemia receiving an echinocandin as initial therapy [46].

However, prolonged use of echinocandins has led to the emergence of resistance mechanisms, especially in *Nakaseomyces glabratus* [50]. The most studied mechanism involves point mutations in conserved regions (hotspots) of the *FKS1, FKS2* and *FKS3* genes, reducing drug affinity for the target enzyme [51,52]. Although mutations in *FKS3* have been reported, this gene is weakly expressed or not at all in most *Candida* species. Among the less susceptible yeast species, resistance to echinocandins has been reported in approximately 2–13% of *N. glabratus* isolates and 0–8% of *C. auris* [35,53]. Species such as *Candida parapsilosis*, *Meyerozyma guilliermondii* (formerly *Candida guilliermondii*), and the *Candidozyma haemuli* (formerly *Candida haemulonii*) complex display reduced susceptibility, and certain species such as *C. parapsilosis* and *M. guilliermondii* exhibit intrinsically higher MICs to echinocandins due to natural polymorphisms in their *FKS* genes. These inherent alterations result in reduced susceptibility despite the conserved mechanism of action. In addition, caspofungin shows the paradoxical growth effect (PGE) at high concentrations, particularly in filamentous fungi like *Aspergillus* [54].

These mutations, either acquired or intrinsic, represent the primary mechanism of echinocandin resistance. The majority of mutations described in *Candida*, *Aspergillus*, and *Cryptococcus* isolates with acquired resistance occur in hotspots of the *FKS1* gene, which is essential in *C. albicans*, whereas in *N. glabratus* both *FKS1* and *FKS2* are viable and mutations may affect either of them due to their functional redundancy. Acquired mutations have been documented in *C. albicans*, *Candida tropicalis*, *Pichia kudriavzeveii* (formerly *Candida krusei*), and *N. glabratus*, while *C. parapsilosis* and related species (*Candida orthopsilosis*, *Candida metapsilosis*) harbor a natural P660A substitution in *FKS1* hotspot 1 that confers intrinsically reduced susceptibility [55]. In *C. auris*, resistant strains with substitutions in the *FKS* subunits have been described, some of which may arise after exposure to echinocandins in animal models [56]. Resistance has also been documented in the *C. haemuli* complex, with mutations such as R1354H in hotspot 2 of *FKS1*, and in *M. guilliermondii*, which has a higher rate of spontaneous mutations in *FKS1*, suggesting intrinsic resistance [56,57]. In *Aspergillus*, mutations in *FKS* homologous genes associated with echinocandin resistance have been identified, including Ser678 substitutions in *A. fumigatus* and Arg1385 substitutions in *Aspergillus nidulans* [58,59,60].

Beyond genetic mutations, genomic plasticity (e.g., chromosome 2 trisomy in *C. albicans*) may confer tolerance, and *ERG3* mutations in *N. glabratus* and *C. auris* can generate cross-resistance to fluconazole [61,62]. Fungi also activate compensatory pathways (PKC-MAPK, Ca^2+^/calcineurin, HOG) that promote cell wall repair via chitin and mannan upregulation, contributing to drug tolerance [13,46,63]. Functional mechanisms such as glucan synthase overexpression and biofilm formation further reduce β-glucan dependence, leading to therapeutic failure even without *FKS* mutations. Cross-resistance among echinocandins is common, limiting treatment options [53].

### 2.3. Polyene

Polyene macrolides, including amphotericin B, nystatin, and natamycin, represent a cornerstone in the treatment of invasive fungal infections, particularly in immunocompromised patients. Their discovery in the mid-20th century marked a milestone in antifungal therapy, and they remain clinically relevant due to their broad spectrum of activity—including multidrug-resistant pathogens such as *C. auris*—and their fungicidal efficacy.

The classical mechanism of action of polyenes relies on their high affinity for ergosterol. These polyene molecules, characterized by multiple conjugated double bonds, insert into the lipid bilayer and bind ergosterol, leading to membrane permeability alterations either by ergosterol sequestration or by forming transmembrane pores. These channels allow uncontrolled flux of ions such as potassium, sodium, and protons, resulting in osmotic imbalance, cell lysis, and death [64,65,66]. In addition to this classical pore-forming model, evidence also supports the “sterol sponge” model, in which amphotericin B primarily exerts its antifungal activity by extracting and sequestering ergosterol from the fungal lipid bilayer, thereby disrupting membrane integrity and function [67]. This mechanism explains their fungal selectivity, although partial interaction with human cholesterol accounts for systemic toxicity, particularly the nephrotoxicity associated with amphotericin B. Additional modes of action have been proposed for amphotericin B, including induction of apoptosis, oxidative damage, and disruption of membrane-associated protein function, compromising cellular homeostasis and suggesting a more complex fungicidal activity [66].

Polyene antifungals exhibit a broad spectrum of activity, encompassing yeasts such as *Candida* (including azole-resistant strains) and *C. neoformans*; filamentous fungi such as *Aspergillus* and *Mucorales*; and dimorphic fungi including *Histoplasma capsulatum*, *Blastomyces dermatitidis*, and *Coccidioides* [68]. Nystatin is primarily used topically due to systemic toxicity, whereas amphotericin B is used to treat invasive fungal infections [69]. However, the availability of alternative antifungal drugs and its toxicity have reduced its use for common systemic mycoses, such as candidiasis and aspergillosis. To mitigate toxic complications, lipid-based formulations of amphotericin B have been developed, improving safety without compromising efficacy, such as amphotericin B lipid complex and liposomal amphotericin B. Nevertheless, several studies indicate that treatment of systemic mycoses caused by species such as *C. auris*, *Aspergillus terreus*, *Scedosporium apiospermum* or *Lomentospora prolificans* may not always be effective, often due to intrinsic or acquired resistance [68].

Although historically considered rare, resistance to polyene antifungals has emerged as a clinical challenge due to increasing infections by non-*Candida albicans* yeasts and resistant filamentous fungi. Amphotericin B, the main representative of this class, maintains a low overall resistance rate, but documented cases in *C. auris*, *N. glabratus*, and *A. fumigatus* highlight the genetic plasticity of these pathogens and their ability to adapt under drug pressure [13].

The most characteristic mechanism of polyene resistance involves alterations in the sterol biosynthesis pathway, reducing or eliminating ergosterol from the fungal membrane—the primary drug target—as well as lipid composition adaptations that decrease drug affinity [66]. Mutations in genes such as *ERG2*, *ERG3*, and *ERG6* modify sterol structure, reducing the binding affinity of amphotericin B and nystatin. This phenomenon has been described in *C. albicans* and *C. tropicalis* and is associated with ergosterol replacement by intermediate sterols, conferring resistance without compromising cell viability, although often accompanied by a measurable reduction in fungal fitness, including impaired growth and attenuated virulence [35]. In *C. auris*, chromosomal rearrangements and mutations affecting lipid homeostasis have been identified, along with epigenetic mechanisms regulating ergosterol pathway gene expression [70].

In filamentous fungi such as *A. fumigatus*, acquired resistance to amphotericin B has been linked to variations in genes involved in sphingolipid synthesis and transmembrane transport. Genome-wide association studies have identified insertions/deletions in *AFUA_7G05160*, encoding a fumarylacetoacetate hydrolase family protein, correlated with polyene resistance. These changes alter membrane composition and reduce the ability to form pores of the drug [71]. Additionally, intrinsically reduced susceptibility has been observed in *Mucorales* species and *A. terreus*, limiting amphotericin B efficacy in mucormycosis and invasive aspergillosis [72].

Clinical resistance, understood as therapeutic failure, including cases of clinical recalcitrance or non-responsiveness despite in vitro susceptibility, does not always correlate with increased minimum inhibitory concentrations (MICs). Factors such as host immunosuppression, PK/PD parameters, and tissue localization influence treatment outcomes. However, the emergence of strains with stable genetic resistance poses a significant threat to the future efficacy of polyenes, particularly in hospital settings where multidrug-resistant isolates are reported [14].

### 2.4. 5-Fluorocytosine

5-fluorocytosine, or flucytosine, is a pyrimidine antimetabolite that acts as a prodrug, whose antifungal activity depends on its intracellular conversion into toxic metabolites. The drug enters the fungal cell through cytosine-specific permeases (Fcy2), which are absent in human cells and therefore confer selectivity. Once inside, cytosine deaminase (Fcy1) converts 5-fluorocytosine into 5-fluorouracil, which is subsequently transformed by uracil phosphoribosyltransferase (Fur1) into fluorinated nucleotides. These metabolites exert two main effects: inhibition of RNA synthesis, through the incorporation of 5-fluorouracil into RNA, altering its processing and ribosomal function, and inhibition of DNA synthesis, through the formation of 5-fluorodeoxyuridylate, which blocks thymidylate synthase and prevents thymidine synthesis. The combination of these mechanisms leads to disruption of protein synthesis and cell death [73,74].

The spectrum of activity of 5-fluorocytosine includes high efficacy against *Candida* species and related yeast, except *P*. *kudriavzevii*, which exhibits intrinsic resistance due to inherently low cytosine deaminase activity and limitations in drug uptake, and against *C. neoformans*, including cases of cryptococcal meningitis. In *Cryptococcus*, hypermutation phenomena that facilitate rapid resistance development are often associated with defects in DNA mismatch repair pathways, particularly mutations in *MSH2* [75]. However, 5-fluorocytosine lacks activity against filamentous fungi such as *Aspergillus* and *Mucorales* [74,76]. In clinical practice, 5-fluorocytosine is mainly used in combination with amphotericin B for the treatment of cryptococcal meningitis and invasive candidiasis, which reduces the emergence of resistance and improves early fungicidal activity [74]. Monotherapy with 5-fluorocytosine is avoided because resistance can emerge rapidly, even during short treatment courses.

Resistance to 5-fluorocytosine is associated with mutations in the *FCY1*, *FCY2*, and *FUR1* genes, which reduce drug activation. Additionally, alterations in nucleotide synthesis pathways and epigenetic mechanisms, such as tRNA modifications, have been described as contributing to resistance. Recent studies have documented clonal resistant expansions in *C. tropicalis* and hypermutation phenomena in *Cryptococcus* [77,78]. Similarly, in *C. auris*, truncating mutations in *FUR1* and deletions in *FCY2* have been identified after few exposures to the drug, demonstrating how rapidly resistance can emerge under selective pressure [79]. Epigenetic and adaptive mechanisms involving global transcriptomic and proteomic regulation changes may facilitate transient resistance that, under selective pressure, becomes consolidated through stable mutations.

From an epidemiological perspective, the species most frequently associated with 5-fluorocytosine resistance are *C. albicans*, *C. tropicalis*, *N. glabratus*, and *C. auris*, as well as *C. neoformans*. The intrinsic resistance of *P. kudriavzevii* is considered primary and is related to its inherently low cytosine deaminase activity and limited drug transport capacity.

### 2.5. Allylamines

Allylamines represent a widely used class of synthetic antifungal agents, which include terbinafine and naftifine. Like azoles, they target the ergosterol biosynthetic pathway but act at an earlier step by inhibiting the endoplasmic reticulum-associated enzyme squalene epoxidase, encoded by the *ERG1* gene, which catalyzes the conversion of squalene to 2,3-oxidosqualene [80]. Inhibition of this reaction leads to intracellular accumulation of squalene and depletion of ergosterol, ultimately compromising membrane integrity and causing fungal cell death.

Allylamines exhibit fungicidal activity against dermatophytes and fungistatic activity against *C. albicans* [81]. However, their activity against other *Candida* species and against filamentous fungi is generally limited, which further explains their primary clinical focus on dermatophytoses. Clinically, they are widely used in superficial mycoses such as tinea corporis, tinea cruris, tinea pedis, cutaneous candidiasis, pityriasis versicolor and onychomycosis, the latter particularly associated with terbinafine [82,83]. Terbinafine can be administered orally or topically. Topical application is often preferred due to direct deposition at the site of infection, lower systemic exposure and minimal toxicity. The drug is highly lipophilic, poorly water-soluble and exhibits high plasma protein binding [84].

In recent years, resistance to allylamines has emerged as a significant clinical concern, particularly within the *Trichophyton* genus (*T. rubrum*, *T. mentagrophytes* and *T. interdigitale*) [85]. Single-nucleotide variations in *ERG1*, most notably Leu393Phe, Leu393Ser and Phe397Leu, have been repeatedly associated with high terbinafine MICs [86]. These substitutions are located near the terbinafine-binding pocket and significantly reduce drug affinity. Beyond target alterations, resistance may involve up-regulation of efflux pumps, including ABC transporters (*PDR1*, *MDR2*, *MDR3*, and *MDR4*) and the plasma membrane ATPase Pma1, which reduce intracellular drug concentrations and may act synergistically with *ERG1* mutations [87,88,89]. Additional mechanisms such as overexpression of downstream ergosterol biosynthesis genes, heat-shock responses and biofilm formation further contribute to reduced terbinafine susceptibility [90,91].

### 2.6. Triterpenoids

Ibrexafungerp represents the first new class of antifungal agents, triterpenoids, approved by the USA Food and Drug Administration (FDA) in 2021 for the treatment of vulvovaginal candidiasis and other fungal infections [92]. It inhibits fungal β-1,3-glucan synthase, similarly to echinocandins, resulting in a low risk of off-target effects; however, unlike echinocandins, it is orally administered. Ibrexafungerp exhibits broad in vitro activity against several *Candida* species, including azole- and echinocandin-resistant *C. albicans* and fluconazole-resistant *C. auris* isolates [93,94,95]. Activity has also been demonstrated against *Aspergillus* spp., including azole-resistant strains of *A. fumigatus* [96], and *Mucorales* [97]. Although currently approved only for vulvovaginal candidiasis, it is under clinical investigation for invasive fungal infections and holds potential for use in hospitalized patients, including those with invasive candidiasis and resistant aspergillosis [3].

## 3. New Strategies to Overcome Antimicrobial Resistance Mechanisms

The increasing challenge of antimicrobial resistance, particularly in fungal infections, has driven the development of several novel strategies focused primarily on the discovery of novel antifungal agents, advanced drug delivery systems and combination therapies. The new strategies to overcome antimicrobial resistance mechanisms fall predominantly into these categories:

### 3.1. Novel Antifungal Agents

The discovery of novel antifungal compounds involves either enhancing current drug classes or exploring new targets, such as the calcineurin pathway (a compensatory pathway mentioned in Section 2), glycosylphosphatidylinositol (GPI) anchor biosynthesis, or farnesyltransferase inhibition [98,99,100,101]. Fosmanogepix is another novel antifungal agent, still in clinical trials, that inhibits an enzyme called Gwt1, which is involved in the biosynthesis of glycosylphosphatidylinositol anchors, crucial for cell wall consistency. It exhibits broad-spectrum activity against yeasts, including *Cryptococcus* and *Candida*, as well as filamentous fungi [102]. Another successful example of a new antifungal agent is olorofim, a first-in-class member of the orotomides, which utilizes a new mechanism of action by reversibly inhibiting dihydroorotate dehydrogenase, a key enzyme that catalyzes the fourth step in the de novo synthesis of pyrimidine nucleotides in DNA synthesis [103,104]. The spectrum of activity of olorofim is unique because of the phylogeny of the targeted enzyme: it has activity against a variety of mold species and thermally dimorphic fungi but lacks activity against yeasts, including *Candida* and *Cryptococcus*, as well as members of the order *Mucorales* [104]. It has been proven active in vitro against azole-resistant *Aspergillus* species, *Scedosporium* species, *Lomentospora prolificans*, which is pan-resistant to clinically available antifungals, and *Microascus* and *Scopulariopsis* species, which are generally considered to have reduced susceptibility or resistance to currently available antifungal agents [103,105,106,107,108].

### 3.2. Plant-Derived Products

Extensive study of natural products with antifungal activity and their biosynthesis, along with chemical probing methods and compound libraries, is ongoing due to their diverse mechanisms of action. Plants, for instance, have evolved alongside phytopathogenic fungi, developing natural defense against these pathogens. Some plant-derived essential oils have demonstrated the ability to inhibit fungal cytochrome P450, increase membrane permeability, block cell wall formation, induce mitochondrial dysfunction, and inhibit efflux pumps and biofilm formation [109,110,111,112]. However, cytochrome P450 inhibition appears to be a secondary consequence of the primary non-specific disruption of fungal membrane integrity rather than a highly selective molecular interaction. Interestingly, combining plant-derived products with other antifungals can trigger synergistic effects resulting in improved efficiency of the drug against resistant strains [109]. Recent work using liposomes composed of dimethyldioctadecylammonium bromide (DDA) and monoolein (DDA:monoolein) to encapsulate carvacrol, thymol, citral, and cinnamaldehyde has demonstrated high encapsulation efficiencies, low cytotoxicity, and enhanced anti-*Candida* activity, including improved macrophage-mediated killing, highlighting the therapeutic potential of lipid-based nanocarriers for essential oils [113].

### 3.3. Antimicrobial Photodynamic Therapy (aPDT)

Antimicrobial photodynamic therapy (aPDT) has emerged as a promising approach as a broad-spectrum antimicrobial treatment [114], including resistant fungal species [115]. It combines visible or near-infrared light, molecular oxygen and a photosensitizer molecule that absorbs the radiation and transfers it to oxygen to generate reactive oxygen species (ROS) that have cytotoxic effects. This therapy induces non-specific oxidative damage, which limits the development of stable resistant mechanisms, and has shown excellent potential for treating biofilm-associated infections [116,117].

### 3.4. Nanotechnology-Based Approaches

The rapid advancement of nanotechnology has opened an expansive range of solutions to critical challenges across numerous fields, including drug delivery and antimicrobial resistance [118]. Nanocarriers provide unique strategies to circumvent complex cellular and physical defense mechanisms employed by resistant fungi. The encapsulation of active substances in drug delivery systems (DDSs) is transforming antifungal treatment by enhancing bioavailability, stability, drug release kinetics, residence time, targeted delivery that increases drug concentration inside fungal cells, and overall efficacy of encapsulated compounds [119].

Among different DDSs, this review focuses on lipid-based drug delivery systems (LDDSs) including, among others, liposomes (LPs), lipid nanoparticles (LNPs), solid lipid nanoparticles (SLNs) and nanostructured lipid carriers (NLCs), as they present optimal characteristics. They successfully overcome multiple intrinsic limitations associated with conventional drug formulations, particularly high toxicity, poor bioavailability, instability, and, crucially, antimicrobial resistance mechanisms (as discussed in Section 4), for instance by promoting direct fusion with fungal cell membranes and bypassing efflux pump-mediated drug extrusion. While many nanocarriers can carry drugs, lipid nanoparticles stand out by combining high versatility (encapsulating lipophilic and hydrophilic drugs) with demonstrated lower toxicity, superior structural integrity, and proven manufacturability [120,121,122]. Their advantages stem from their physicochemical composition (biocompatible lipids) and their nanometric scale, which provides them with unique kinetic and targeting capabilities (Table 1).

#### 3.4.1. Liposomes (LPs)

Liposomes (LPs) are a significant class of lipid-based drug carriers that marked the beginning of advanced lipid nanoparticle delivery systems. They were first described in 1964 [123], and since then, numerous studies have led to important technical advances in their application. They are recognized as the first lipid-based nanomedicine delivery system to progress from a laboratory concept to clinical application, achieving high clinical acceptance [124,125].

LPs are spherical vesicles composed of amphipathic lipid bilayers. The bilayer can be single (unilamellar vesicles, ULVs), including small, large, and giant unilamellar vesicles (SUVs, LUVs, and GUVs, respectively), or multiple (multilamellar vesicles, MLVs), forming an aqueous core in which hydrophilic drugs can be encapsulated. Their size can range from 20 nm to several micrometers, depending on the preparation method. Small unilamellar vesicles are the simplest form, consisting of a single lipid bilayer and typically measuring between 20 and 100 nanometers. They are relatively easy to produce and are useful for drug delivery, although their internal volume is limited. Large unilamellar vesicles also have a single bilayer but are bigger, which allows them to encapsulate larger amounts of hydrophilic drugs. In contrast, multilamellar vesicles contain several concentric lipid bilayers arranged like an onion. These structures tend to be more stable but release their contents less uniformly. Giant unilamellar vesicles are much larger and are mainly used in experimental research as model systems for biological membranes rather than in clinical applications. Typical components of LPs include (i) phospholipids that provide the main structure (e.g., phosphatidylcholine, phosphatidylserine, phosphatidylethanolamine), (ii) cholesterol, which modulates bilayer fluidity, stability, and permeability, and (iii) optional surface modifiers that influence biodistribution and targeting (i.e., polyethylene glycol, ligands, or antibodies). Hydrophilic drugs are mainly entrapped in the aqueous core, whereas hydrophobic compounds are incorporated within the lipid bilayer. In the context of antifungal delivery, such as amphotericin B, it is worth emphasizing that cholesterol and other sterols play a crucial role in stabilizing the drug within the liposomal membrane by preferential drug–sterol interactions, thereby reducing the free drug fraction and systemic toxicity.

LPs, as a class of lipid-based vesicular nanocarriers, primarily overcome antifungal resistance by enhancing drug efficacy, improving drug stability, mitigating dose-limiting toxicity [126] and facilitating cellular uptake via membrane fusion or endocytosis, thereby circumventing fungal defense mechanisms like efflux pumps [127].

Modern advancements in lipid nanoparticle design, including modifications applicable to LPs, enhance their ability to target fungal cells and overcome resistance barriers.

#### 3.4.2. Solid Lipid Nanoparticles (SLNs)

Solid lipid nanoparticles (SLNs) represent a promising and robust class of nanocarriers for controlled drug delivery. They consist of a hydrophobic inner core made of lipids surrounded by an outer stabilizing layer composed of amphiphilic surfactants and, in some cases, co-surfactants [128,129].

SLNs are characterized by being formulated from physiological and biocompatible lipids. Their excipients are often materials generally recognized as safe (GRAS), which contribute to their low inherent toxicity and facilitate regulatory acceptance.

The key structural feature that distinguishes SLNs from other lipid-based nanocarriers is that the lipid matrix remains solid at both room temperature and human body temperature. This property makes SLNs particularly suitable for controlled and sustained drug delivery, as the drug slowly diffuses through the solid lipid matrix, resulting in prolonged release profiles. Therefore, the use of SLNs reduces dosing frequency and improves therapeutic efficacy.

Optimized SLNs are typically spherical in shape with a smooth surface. They have a diameter between 50 and 1000 nm, but as discussed in Section 5.2, most pharmaceutical formulations are around 50–300 nm because small size favors enhanced penetration, cellular uptake, and biodistribution.

Compared to LPs or emulsions, SLNs present improved physical stability and are generally less prone to drug leakage, particularly for hydrophilic compounds. The solid state of lipids also contributes to enhanced shelf stability and prolonged storage capabilities. However, for lipophilic drugs, a known limitation of SLNs is the potential for drug expulsion during storage, as the solid lipid matrix may undergo a transition to a highly ordered crystalline state that reduces the space available for drug molecules and limits drug-loading capacity. Another relevant feature for drug delivery is their high versatility across administration routes, as they are suitable for oral, topical, dermal, ocular, nasal, intravenous, and pulmonary delivery [130]. Despite this limitation, SLNs can better maintain drug integrity and ensure effective delivery to fungal cells, making them promising carriers for improving cellular penetration and reducing the impact of resistance mechanisms mediated by efflux pumps or biofilms [131].

#### 3.4.3. Nanostructured Lipid Carriers (NLCs)

Nanostructured lipid carriers (NLCs) are considered second-generation SLNs, as they retain the advantages of SLNs while offering a significantly higher drug loading capacity [121]. This improvement arises from their matrix composition, which consists of a blend of solid lipids and liquid lipids (oils) rather than a purely solid lipid core. Common solid lipids include glyceryl behenate and stearic acid, while liquid lipids often include oleic acid, castor oil, or caprylic/capric triglycerides [132]. Depending on the lipid composition and preparation method, NLCs can be classified into three structural models: imperfect, amorphous, and multiple-type NLCs. These architectures generate a less ordered crystalline structure, as the incorporation of liquid lipids disrupts lipid packing and prevents the solid lipid matrix from transitioning into a highly ordered β-crystalline form, which is primarily responsible for drug expulsion during storage. Reduced crystallinity thereby minimizes drug leakage, enhancing physical stability and resulting in improved long-term storage stability [133].

Apart from the inner core characteristics and their impact on drug loading capacity and storage stability, SLNs and NLCs present similar physicochemical characteristics, including size, surface characteristics, biocompatibility, controlled drug release and manufacturing versatility.

#### 3.4.4. Lipid Nanoparticles (LNPs)

Lipid nanoparticles (LNPs) are multicomponent supramolecular assemblies with complex structural and morphological features composed of lipid bilayers or micellar-like structures. They are composed of amphiphilic lipids (mainly phospholipids), cholesterol, and very often PEGylated lipids for stealth purposes. As they are especially optimized for nucleic acid delivery, they typically present ionizable or cationic lipids in their composition [134]. The components form an amorphous lipid matrix capable of efficiently encapsulating and protecting cargo (i.e., labile genetic material) with excellent loading capacity. With particle sizes typically ranging from 60 to 150 nm, LNPs exhibit favorable pharmacokinetics and efficient cellular uptake (high transfection efficiency). In addition, they present scalable and reproducible manufacturing via microfluidic mixing. These characteristics have established LNPs as the primary delivery system for mRNA vaccines and emerging gene therapies, and some formulations have already been clinically validated [135].

Nonetheless, LNPs are less suitable for hydrophobic small-molecule drugs, present stability issues during long-term storage, and, due to their complex formulation, entail higher production costs compared to SLNs/NLCs [136]. However, despite these limitations, LNPs hold considerable potential as delivery platforms for nucleic acid-based therapeutics, such as siRNA or antisense oligonucleotides, enabling the silencing of fungal genes associated with antifungal resistance (e.g., *ERG11*) and thereby offering an alternative strategy to overcome resistance mechanisms [137].

## 4. Lipid-Based Drug Delivery Systems for Combating Antifungal Resistance

**Figure 1:**
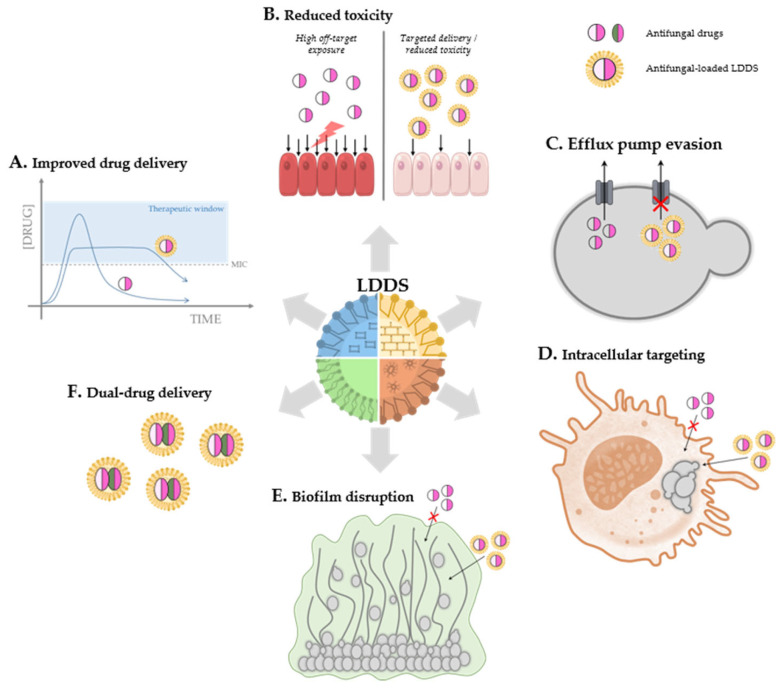
Mechanisms by which lipid-based drug delivery systems (LDDSs) contribute to overcoming antifungal resistance. LDDSs—NLC (blue), SLN (yellow), LP (green) and LNP (brown)—improve antifungal therapy through multiple complementary mechanisms. By (**A**) enhancing drug solubility, stability, protection, bioavailability, and controlled release, these systems maintain effective drug concentrations and reduce selective pressure for resistance. Lipid nanocarriers can also (**B**) reduce drug toxicity, enabling higher or prolonged therapeutic dosing. In addition, LDDSs may (**C**) circumvent efflux pump-mediated resistance, (**D**) enhance intracellular delivery to infected host cells, and (**E**) improve penetration into fungal biofilms. The possibility of (**F**) co-delivering multiple active compounds within a single nanocarrier further enables synergistic antifungal strategies that target multiple resistance pathways simultaneously.

Lipid-based drug delivery systems (LDDSs) provide multiple strategies to prevent, mitigate, and counteract antifungal resistance [138,139,140,141] (Figure 1). Their primary role is widely recognized to be the enhancement of drug delivery, therapeutic efficacy, and safety profiles of the therapeutic agents they transport [139,141]. Within the context of antifungal resistance, these advantages translate into an improved ability to overcome resistance mechanisms and effectively treat infections caused by resistant strains. LDDSs can address key resistance mechanisms described in Section 2 by enhancing intracellular drug accumulation and bypassing efflux pump-mediated drug extrusion, promoting penetration into fungal biofilms and disrupting their protective extracellular matrix, and improving drug access to intracellular or poorly accessible fungal reservoirs [138,141,142,143]. Finally, the capacity of LDDSs to co-encapsulate more than one therapeutically active compound—such as combining an azole with synergistic natural products against azole-resistant *C. albicans*—positions these delivery systems as an appealing platform for combination strategies aimed at reducing the emergence and persistence of antifungal resistance [109,144,145].

### 4.1. Enhancing Drug Delivery and Efficacy

The most widely described strategy by which LDDSs contribute to overcoming antifungal resistance is by modulating the pharmacokinetic behavior of the encapsulated drug, improving its effective delivery through enhanced stability, solubility, bioavailability, and release profiles (Figure 1A). LDDSs protect incorporated active compounds from both chemical and biological degradation, enhancing their stability during storage and administration [141,146]. They shield drugs from environmental factors such as pH and temperature fluctuations, light exposure, oxygen, and moisture [138,147]. This is particularly relevant for unstable compounds like essential oils, including carvacrol, thymol, citral, and cinnamaldehyde, which are effectively stabilized against oxidation and volatility [113,148,149]. LDDSs also offer protection against enzymatic degradation by host enzymes or those produced by pathogens [138,150]. LDDSs additionally protect encapsulated compounds from host inflammatory responses, further contributing to the preservation of their structural integrity and biological activity [150,151]. This shielding ensures that the antimicrobial drug remains active until it reaches the site of infection, preventing premature degradation that could result in low effective concentrations and selective pressure favoring resistant strains.

Besides protection, LDDSs are highly effective in encapsulating poorly water-soluble drugs such as polyenes and certain azoles (e.g., itraconazole and ketoconazole), significantly increasing their aqueous solubility and enabling their formulation in dispersed aqueous systems [146,152]. This solubility enhancement ensures that sufficient concentrations of the drug are available for absorption and therapeutic action [153]. Improved solubility not only enhances absorption but also minimizes the risk of subinhibitory concentrations, which are strongly associated with resistance development. For example, oral administration of ketoconazole-loaded SLNs and NLCs showed a significant improvement in bioavailability compared to conventional suspensions, resulting in enhanced antifungal efficacy against *C. albicans* [154]. Similarly, encapsulation of amphotericin B results in high oral bioavailability, enabling oral administration in mice [155]. This represents a major breakthrough for a drug that is traditionally limited to intravenous use due to poor gastrointestinal absorption, as oral LDDS formulations could substantially improve patient compliance and reduce the need for prolonged hospital stays. Moreover, oral LDDS administration can bypass presystemic metabolism by promoting chylomicron formation and lymphatic transport, thus avoiding first-pass hepatic metabolism and maximizing drug exposure [156].

Moreover, the lipophilic nature and small particle size of LDDSs allow efficient penetration through biological barriers—including skin, mucosa, and corneal layers—and selective uptake by macrophages and other target cells, thereby concentrating the drug at the site of infection and significantly improving therapeutic efficacy [146,157]. Importantly, macrophage-mediated uptake enables LDDSs to target intracellular fungal reservoirs, a key strategy for controlling persistent infections and preventing chronic and resistant infections, as further discussed in Section 4.3.2. This site-specific targeting maximizes antimicrobial action while minimizing exposure to non-target tissues [153]. Nanoencapsulation may also facilitate enhanced penetration into fungal cells via adsorption to and fusion with the fungal cell membrane, promoting closer interaction with the cell wall and membrane and reinforcing mechanisms such as ergosterol inhibition and membrane disruption, which have been associated with increased antifungal activity against *Candida* [158,159]. Additionally, the solid or semi-solid lipid matrix of SLNs and NLCs enables sustained and controlled release, generating a local depot and maintaining therapeutic concentrations over extended periods [146,152]. This controlled release minimizes the “burst effect”, reduces dosing frequency, and ensures continuous pathogen exposure to the antimicrobial agent, which is essential to prevent the emergence of resistant subpopulations [160].

Through the optimization of these pharmacokinetic properties, lipid nanoparticles influence drug pharmacodynamics, ensuring that pathogens are consistently exposed to effective drug concentrations and thus reducing the selective pressure that drives antimicrobial resistance.

### 4.2. Enhancing Safety and Tolerability

One of the major advantages of lipid nanocarriers is their ability to reduce the intrinsic toxicity of antifungal drugs while improving overall tolerability (Figure 1B). Many conventional antifungal agents—particularly amphotericin B and several azoles—are limited by dose-dependent adverse effects and off-target interactions that often compromise patient compliance [138,141]. This improved safety profile enables the use of otherwise highly toxic compounds, thereby expanding the therapeutic options available against resistant pathogens and supporting prolonged or higher-dose treatments required to address antifungal resistance [138,141]. Through the reduction in systemic toxicity associated with conventional antifungals, lipid nanocarriers also enable sustained and localized administration at higher drug concentrations at the site of infection, an important advantage in settings where resistance limits therapeutic efficacy [161].

A key determinant of this improved safety profile is the intrinsic biocompatibility of lipid excipients, many of which are classified as GRAS. This regulatory status substantially lowers the barrier for clinical translation and regulatory approval compared to novel synthetic polymers, as these excipients are already well characterized for human use. These excipients resemble physiological lipids, are biodegradable, and are generally well tolerated. Numerous studies demonstrate that SLNs, NSCs, LPs, LNPs and nanoemulsions show minimal cytotoxicity and favorable tolerability in vitro and in vivo [132,138,140].

The most widely recognized example of toxicity reduction achieved through lipid carriers is amphotericin B. Lipid-based formulations reduce nephrotoxicity by modifying the drug interaction with host cell membranes while preserving or even enhancing antifungal activity [153,162,163]. Amphotericin B toxicity is strongly determined by its aggregation state: dimers and polyaggregates induce mammalian cell damage, whereas monomers or small aggregates selectively bind ergosterol in fungal membranes [153,162,163]. In lipid-based formulations, particularly LPs, the lipid environment acts as a membrane mimic that stabilizes amphotericin B in its monomeric or low-aggregation state, preventing the formation of toxic polyaggregates. Consequently, lipid formulations improve selective antifungal activity while reducing off-target toxicity [153,163]. Consistently, amphotericin B-loaded SLNs, NLCs, LPs, LNPs, and nanoemulsions have shown reduced hemolysis, decreased renal accumulation, and improved tolerability in preclinical models [150,164].

Comparable benefits have been reported for azole-loaded lipid carriers. Encapsulation reduces mucosal irritation and systemic adverse effects associated with high drug levels [161,165]. Lipid delivery systems enable localized drug delivery and limit or even eliminate systemic absorption, which is particularly beneficial for azoles such as voriconazole that are associated with hepatic toxicity, visual disturbances, nausea, and gastrointestinal discomfort when administered systemically [166,167,168,169]. By enabling topical or ocular administration, LDDSs can bypass systemic exposure entirely while maintaining high local drug concentrations at the site of infection, representing a key advantage for the treatment of localized and potentially resistant fungal infections. Moreover, lipid carriers often enable therapeutic efficacy at lower drug doses [170,171,172].

Finally, it is important to highlight that the improved safety of lipid nanocarriers is strongly linked to their ability to modulate drug pharmacokinetics. Enhanced bioavailability and sustained, controlled release help avoid the sharp plasma concentration peaks associated with the toxicity of conventional formulations [147,173]. By maintaining prolonged therapeutic levels, these systems allow dose reduction and less frequent administration [159,174], ultimately improving patient adherence [141].

### 4.3. Overcoming Resistance Mechanisms

One of the most critical challenges in antimicrobial therapy is the development of resistance through diverse microbial strategies. Lipid nanoparticles have emerged as versatile platforms capable of overcoming resistance mechanisms by inhibiting drug efflux pumps, targeting intracellular pathogens, and disrupting biofilm formation.

#### 4.3.1. Efflux Pump Inhibition

Efflux pumps are among the most common mechanisms of antifungal resistance. Fungal pathogens—particularly *Candida* species—upregulate cell membrane transporters, including ATP-binding cassette (ABC) carriers, which actively expel antifungal drugs and reduce their intracellular concentration below therapeutic levels [142,175]. This mechanism severely compromises clinical outcomes and is considered a major hurdle for effective antifungal therapy [142]. Due to the fact that efflux-mediated resistance reduces intracellular exposure to the drug, strategies that enhance cellular uptake or avoid transporter recognition are essential for antifungal susceptibility recovery.

LDDSs have emerged as a promising strategy to counteract efflux-mediated resistance by enhancing drug entry into fungal cells, protecting the drug from recognition by efflux systems, and increasing intracellular accumulation (Figure 1C) [175,176,177]. Importantly, LDDSs can bypass efflux pumps through endocytic uptake or membrane fusion, enabling the antifungal agent to be released directly into the cytoplasm and effectively circumventing transporters located in the plasma membrane. Accordingly, there is evidence supporting the ability of LDDS formulations to reverse azole resistance in *Candida* spp., which frequently results from the overexpression of cell membrane transport proteins (efflux pumps) that actively expel azoles from the cell [175]. Moazeni et al. developed fluconazole-loaded SLNs and NLCs that restored susceptibility in fluconazole-resistant *C. albicans*, *C. parapsilosis*, and *N. glabratus* [175,176]. NLCs were particularly effective, acting as a nanoscaled safeguard that reduces drug efflux by shielding the antifungal from transporter recognition and promoting efficient cellular entry through their hydrophobic surface [175].

Besides the effects of the lipid matrix itself, surfactants commonly used in LDDS formulations, such as taurocholate, phospholipids, and polysorbates (commonly known as Tweens), can inhibit efflux transporters, thereby promoting intracellular drug retention and restoring antifungal activity [155,156]. In this context, LDDSs act not only as passive carriers but also as pharmacologically active components that synergize with the encapsulated drug to overcome resistance. This effect may involve transporters such as Cdr1p [176]. Efflux pump inhibition can also be achieved through the encapsulation of antifungal natural products. Essential oils possess intrinsic antifungal mechanisms, including the inhibition of efflux pumps [178]. Their incorporation into lipid nanocarriers not only improves their stability and bioavailability but also enables the practical use of these compounds against resistant strains [178,179].

A complementary strategy involves dual-drug delivery systems designed to include an efflux pump modulator. For instance, docetaxel-ketoconazole SLNs were engineered to overcome multidrug resistance mediated by P-glycoprotein, where the ketoconazole component effectively inhibited the transporter and restored intracellular accumulation of the co-encapsulated drug, which is widely used in cancer therapy [180]. Although this example originates from oncology, the underlying principle is directly translatable to medical mycology, as azole resistance in fungi is similarly driven by the overexpression of ABC efflux transporters, such as Cdr1p and Cdr2p. Accordingly, this strategy highlights the potential of LDDSs to co-deliver antifungal agents with chemosensitizers that directly modulate conserved resistance pathways.

#### 4.3.2. Targeting Intracellular Pathogens

Various fungal pathogens, such as *C. albicans* and *C. neoformans*, can survive and proliferate within host cells, where limited drug penetration and protection from the immune system can contribute to therapeutic failure [177,181]. In this context, the ability of lipid-based nanocarriers to efficiently cross physiological membranes and enter host cells has positioned LDDSs as attractive tools to improve drug delivery and reach intracellular reservoirs (Figure 1D) [177,182]. For example, in oral infections caused by *C. albicans*, which induces its own endocytosis in epithelial cells, SLNs may help deliver antifungal agents directly to the invasion site [181].

Although much of the experimental evidence derives from models of intracellular *Leishmania* parasites (obligate intracellular parasites of macrophages), these findings are highly relevant to systemic fungal infections, as several fungal species are also able to persist and proliferate within macrophages during invasive disease [183]. Amphotericin B-loaded SLNs have demonstrated enhanced efficacy against intracellular *Leishmania* due to improved uptake by macrophages and increased intracellular drug accumulation [182,184]. Likewise, macrophage internalization is crucial for antifungal efficacy in invasive mycoses, as the uptake of amphotericin B-loaded LNPs by macrophages has been shown to correlate with enhanced antifungal activity in vitro, underscoring the relevance of macrophage targeting for pathogens capable of intracellular persistence [185]. In this context, macrophage targeting can be further improved through surface functionalization of nanoparticles with macrophage-specific ligands. Vyas et al. [186] demonstrated that aerosolized surface-modified LPs and emulsomes (hybrid lipid carriers composed of a solid fat core surrounded by phospholipid layers) markedly enhanced the intracellular delivery of amphotericin B, leading to a higher therapeutic efficacy and achieving a 100% survival rate in immunosuppressed rats with pulmonary aspergillosis, far exceeding the efficacy of aerosolized conventional amphotericin B (16.7%). Although *Aspergillus* spp. are primarily considered extracellular pathogens, targeting alveolar macrophages is critical, as inhaled conidia can survive and germinate within these cells, contributing to disease progression and persistence.

In order to improve drug delivery against fungal pathogens that persist within macrophages, a particularly innovative approach involves pH-responsive LNPs engineered to undergo structural reorganization in acidic environments, characteristic of fungal infection sites and macrophage lysosomes [177]. These nanoparticles remain neutral hexosomes at physiological pH but convert into cationic cubosomes in acidic conditions (pH ∼ 5), undergoing a structural reorganization that increases their internal surface area and mucoadhesive properties. This transition confers a positive surface charge that promotes electrostatic interaction and potential fusion with the negatively charged fungal cell wall, facilitating closer carrier-pathogen contact. This approach has been successfully applied against fluconazole-resistant *C. neoformans*, which proliferates within macrophage lysosomes. Using this strategy, fluconazole-loaded pH-sensitive LNPs restored antifungal activity against resistant isolates, achieving MIC_90_ values unattainable with the free drug even at high concentrations and overcoming resistance mechanisms such as efflux pumps and target mutations [177].

#### 4.3.3. Biofilm Disruption

Biofilm formation is a major determinant of fungal pathogenicity and antimicrobial resistance, providing protection against both host immune defenses and limiting antifungal drug penetration. Although several fungal species are capable of producing biofilms, *Candida* spp. remains the most prevalent genus associated with biofilm development, which compromises therapeutic efficacy and contributes to recurrent and drug-resistant infections [138,143]. Since Kuhn and colleagues (2002) reported that only lipid formulations of amphotericin B and the echinocandins caspofungin and micafungin were effective against biofilms of *C. albicans* and *C. parapsilosis*, accumulating evidence has demonstrated that lipid nanocarriers are promising tools to both reduce biofilm formation and disrupt mature biofilms through multiple and complementary mechanisms [187].

As previously described, the improved solubility, bioavailability, controlled release, and prolonged residence time provided by encapsulated compounds can help maintain drug concentrations capable of preventing adhesion, hyphal elongation, and extracellular matrix production [170,188,189]. This is particularly important for essential oils that possess intrinsic biofilm-inhibitory activity but are limited by poor stability and solubility; encapsulation into LDDSs effectively overcomes these drawbacks [189,190]. For example, clove oil encapsulated into SLNs shows enhanced antifungal activity against *C. albicans*, likely due to improved bioavailability and effective diffusion through the extracellular polymeric substance (EPS) matrix of microbial biofilms, which facilitates penetration into embedded fungal cells. The nanoscale size and lipophilic nature of SLNs reduce sequestration by biofilm matrix components, resulting in membrane disruption and interference with fungal metabolic processes [189]. Another example is the use of eugenol-loaded NLCs, which reduced the MIC of eugenol against *C. albicans* and *N. glabratus* by up to tenfold and eightfold, respectively, and significantly reduced biofilm formation compared with free eugenol. The increased contact area provided by the nanosized particles, together with their ability to diffuse through the EPS matrix and provide controlled drug release, enhances eugenol-mediated disruption of fungal membrane integrity, ultimately disrupting the biofilm structure [191].

For conventional antifungals encapsulated within lipid formulations, the evidence indicates that LDDSs can enhance activity against established, mature biofilms (Figure 1E). Ketoconazole-loaded SLNs have demonstrated superior efficacy against mature *C. albicans* biofilms compared with the free drug, overcoming biofilm-mediated resistance that increases azole tolerance [170]. Similarly, anidulafungin-loaded LPs and amphotericin B nanoemulsions have shown strong biofilm-disruption activity against *C. auris*, inducing structural damage and achieving up to a 99% reduction in fungal burden within the biofilm [192,193]. These findings are especially relevant given the multidrug-resistant profile of *C. auris*, whose robust biofilm formation represents a major therapeutic challenge [148].

Additionally, emerging strategies explore the synergistic effect of lipid nanocarriers with adjunctive therapies. Hypericin-loaded NLC administered together with aPDT significantly reduced *C. albicans* biofilm viability, highlighting the versatility of lipid nanosystems as delivery platforms capable of integrating multiple therapeutic modalities. NLCs are particularly advantageous for hypericin delivery, as the lipid matrix improves its dispersion and preserves its photodynamically active form in aqueous environments, which is essential for efficient ROS generation upon light irradiation [194].

### 4.4. Dual-Drug Delivery

Dual-drug delivery systems—lipid-based nanocarriers designed to transport two or more active compounds simultaneously—are emerging as a promising strategy to address the growing challenge of antifungal resistance. By targeting multiple cellular pathways at once, co-delivery approaches reduce the likelihood that fungi will develop resistance (Figure 1F) [144,145,195]. These nanosystems also enable drug synergy, dose reduction, and complementary bioactivities that cannot be achieved through monotherapy, especially in infections caused by multidrug-resistant species. A representative example is the co-encapsulation of amphotericin B and micafungin into a single nanoemulsion, which significantly improved therapeutic outcomes in an immunosuppressed mouse model of systemic *C. auris* infection, leading to a marked reduction in fungal burden across multiple organs [196].

In most reported formulations, a conventional antifungal is combined with a natural bioactive molecule—often an essential oil or terpene—to exploit their intrinsic antifungal, membrane-disruptive, or synergistic properties [144,145,195].

Considerable efforts have focused on enhancing azole performance against *Candida* species [144,145]. Carbone et al. demonstrated that co-encapsulating clotrimazole into NLCs containing *Lavandula* or *Rosmarinus* essential oils markedly increased antifungal activity, with up to an eight-fold improvement against *C. parapsilosis*. These effects are attributed to synergistic interactions between the essential oil components and the azole, likely involving membrane perturbation [144]. Subsequent work by the same research group showed that dual loading of clotrimazole and alpha-lipoic acid in SLNs further enhanced efficacy while mitigating oxidative stress, highlighting the added therapeutic value of pairing an antifungal with a cytoprotective molecule [160].

Other studies have explored synergistic combinations involving antifungals and quorum-sensing molecules. Notably, liposomal co-encapsulation of farnesol and fluconazole not only enhanced antifungal activity against *C. albicans* and *C. tropicalis* compared with free farnesol but also converted an antagonistic interaction observed with the free-drug combination into a synergistic effect [197]. This finding highlights that LDDSs do not merely co-deliver multiple agents but can actively modulate their spatial and temporal presentation at the fungal cell interface, thereby reshaping pharmacodynamic interactions. Similarly, bisabolol-fluconazole LPs potentiated fluconazole activity across *C. albicans*, *C. tropicalis*, and *C. krusei*, enabling significant reductions in the required drug concentrations [198]. In another approach, a lipid-nanocarrier gel co-loaded with luliconazole and niacinamide significantly improved antifungal activity in a vaginal candidiasis model, an effect attributed to enhanced membrane permeation and sustained drug release [199].

Beyond conventional lipids, innovative nanocarrier architectures have been explored. A self-assembled pillar [5]arene system functionalized with a farnesyl moiety enabled efficient co-delivery of nystatin, enhancing its antifungal activity against *Candida* and *Saccharomyces* isolates [200]. Likewise, loratadine-loaded invasomes (flexible lipid vesicles containing terpenes such as cineole designed to enhance membrane permeation) leveraged the terpenes’ membrane-disrupting activity to achieve an approximately 3-log reduction in *C. albicans* viability, far surpassing free-drug performance [195]. This example also highlights the potential of lipid-based nanocarriers to facilitate drug repurposing strategies, repositioning non-antifungal agents such as loratadine as effective components of antifungal therapies.

Collectively, current evidence highlights dual-drug lipid nanocarriers as a versatile and powerful platform; their ability to integrate complementary antifungal actions, enhance drug penetration, and sustain therapeutic levels position them as a promising next-generation strategy for managing both resistant and recurrent fungal infections.

## 5. Relevant Nanoparticle Properties That Affect Antifungal Drug Delivery and Efficacy

The performance of lipid-based nanoparticles in antifungal drug delivery is highly dependent on their physicochemical properties. These characteristics determine not only colloidal stability but also biological interactions, tissue penetration, and overall antifungal efficacy. In this section, main LDDS characteristics are discussed.

### 5.1. Composition and Stability

Particle stability is crucial for drug delivery, and it is primarily influenced by the composition of the internal matrix, the characteristics of the particle surface, which is closely related to the selection of surfactants and stabilizers, and the physical state and loading of the encapsulated substance [201].

The selection of solid and liquid lipids of the lipid matrix in SLNs and NLCs is critical for drug incorporation, physical stability, and drug release characteristics. As mentioned before, binary lipid mixtures in NLCs can often accommodate more of a drug and offer better stability than SLNs, which may experience drug expulsion due to lipid recrystallization during storage. The polymorphism of solid lipids (e.g., triacylglycerols like glyceryl tristearate) also influences compound incorporation and release. During storage, lipids tend to transition from metastable polymorphic forms (α or β) to the more stable and highly ordered β-form. This transition is a major driver of drug expulsion in SLNs, as the increasingly ordered structure limits the space available for drug accommodation. Generally, lipids with longer hydrocarbon chain lengths are associated with higher melting points and consequently greater nanoparticle stability. Combining different types of glycerides or lipids can prevent abnormal lipid crystallization, enhancing stability [132].

For LPs, the composition of the lipids is a key determinant [202]. In this context, the Critical Packing Parameter (CPP) of lipid molecules plays a central role in vesicle formation and stability, as it governs the preferred self-assembled structure of amphiphilic lipids. Lipids with CPP values close to unity favor bilayer organization and stable vesicle formation [203]. Accordingly, an optimal proportion of cholesterol and other lipids is required to maintain the integrity and rigidity of liposomal membranes, as cholesterol acts as a membrane stabilizer, increasing vesicle stability and encapsulation efficiency. Cholesterol also reduces membrane permeability, preventing premature leakage of incorporated antifungals [204].

Surfactants and stabilizers are integral components, as they are essential for stabilizing lipid nanoparticles by reducing surface tension and controlling degradation and drug release rates. The hydrophilic-lipophilic balance (HLB) of the surfactant is a key parameter governing colloidal stability, as an appropriate HLB value ensures adequate interfacial coverage and prevents particle aggregation. A suitable surfactant, or the addition of a co-surfactant, therefore plays a key role in avoiding particle aggregation and stabilizing the formulation [205]. The presence of poly(ethylene glycol) (PEG) on the nanoparticle surface can further provide a stealth characteristic by creating a steric barrier that prevents opsonization and subsequent recognition by the mononuclear phagocyte system, thereby increasing blood residence time and reducing toxicity.

### 5.2. Particle Size

The particle size is a critical factor for penetration, biodistribution, cell internalization, and efficacy of nanoparticles [206]. Although smaller nanoparticles exhibit higher surface energy and therefore require adequate stabilization to prevent aggregation, their physical stability in suspension is often enhanced because Brownian motion dominates over gravitational forces, minimizing sedimentation and creaming. Consequently, lipid-based nanoparticles typically present size ranges below 300 nm; however, optimal nanoparticle size ultimately depends on the intended therapeutic goal [207].

For antifungal drug delivery to skin, smaller particles (e.g., less than 200 nm) are generally preferred, as they facilitate enhanced penetration through skin layers (like the stratum corneum) and corneal uptake by epithelial cells [154,157,208]. It has been observed that for cutaneous infections, SLNs between 50 and 200 nm readily penetrate into deep layers of skin, while those between 200 and 400 nm accumulate in the dermis, making both ranges suitable for treating fungal skin infections [209]. Smaller particle size also contributes to the effectiveness of SLNs as ocular antifungal drug delivery systems [210]. Additionally, smaller nanoparticles exhibit a higher surface-to-volume ratio, increasing their interaction with fungal membranes and improving diffusion through biofilms [211]. However, reduced particle size also shortens the diffusion path length within the carrier matrix, which can accelerate drug release and increase the likelihood of an initial burst release compared to larger particles.

### 5.3. Polydispersity Index (PDI)

The polydispersity index (PDI) measures the uniformity of particle sizes within a colloidal system. PDI is a critical indicator of the width of the particle size distribution: values closer to zero reflect a highly monodisperse system, whereas values approaching 1.0 indicate a highly polydisperse population. The PDI is a dimensionless parameter typically derived from Dynamic Light Scattering (DLS) measurements and reflects the breadth of the particle size distribution. Maintaining low PDI values is particularly important for long-term colloidal stability, as high polydispersity can promote “Ostwald ripening”, a process in which larger particles grow at the expense of smaller ones due to differences in solubility, ultimately compromising the stability of the dispersion [212]. In addition, low PDI values indicate good dispersion quality and contribute to improved reproducibility of drug release and tissue penetration. PDI values below 0.3 are generally considered optimal, particularly for parenteral formulations, as regulatory requirements for intravenous administration typically demand narrow size distributions (PDI < 0.2–0.3) to ensure safety, stability, and predictable pharmacokinetics [206]. In contrast, for non-parenteral applications such as topical or oral antifungal formulations, higher PDI values, up to 0.5, are commonly regarded as acceptable and representative of uniform dispersions, as reported for antifungal agents including miconazole, fluconazole, and sulconazole [159,169,213,214].

PDI can be influenced by the concentration of surfactants, as an increase in stabilizer content can sometimes decrease the PDI by preventing the agglomeration of lipid particles [215].

### 5.4. Zeta Potential (ZP)

Zeta potential (ZP) is related to the surface charge of the particles suspended in a liquid and thus reflects the degree of electrostatic repulsion between similarly charged particles, making it a useful parameter for predicting the physical stability of nanoparticle dispersions. A ZP of approximately ±30 mV is generally required for effective electrostatic stabilization, as it prevents particle aggregation through charge-based repulsion [216]. However, in systems stabilized predominantly by steric mechanisms, such as PEGylated nanoparticles, high colloidal stability can be achieved even at ZP values close to zero, since the steric barrier provided by the polymer chains effectively inhibits particle-particle interactions [217].

In addition, surface charge influences interaction with biological membranes and cellular uptake. Thus, the choice between a positive or negative ZP often depends on the biological target. For treating corneal infections, positively charged lipid nanoparticles (often modified with chitosan or stearylamine) are frequently preferred, as they establish electrostatic interactions with negatively charged sialic acid residues in corneal mucins, increasing pre-corneal residence time and improving drug penetration [218]. Positively charged nanoparticles, such as cationic econazole-loaded SLNs (ZP values 19.13 ± 0.89 mV), can show enhanced corneal penetration and ocular bioavailability due to electrostatic interactions with the negatively charged corneal surface [165]. Cationic nanoparticles can also interact with the negatively charged fungal cell wall, facilitating closer contact and improved drug uptake [219]. Although some authors suggest that positively charged nanoparticles can reverse fungal cell surface charge inducing fungal death, antifungal activity is generally attributed to electrostatic disruption and destabilization of the fungal membrane, leading to increased permeability and ultimately cell death [220].

Conversely, negatively charged lipid carriers have also been reported to facilitate drug entry into yeast cells, such as ketoconazole-loaded NLCs (−27.8 to −32.5 mV) [221], miconazole nitrate SLNs (−21.6 to −31.4 mV) [222], and fluconazole-loaded SLNs or NLCs (−25 mV and −20 mV, respectively) [223]. In these systems, it is proposed that hydrophobic interactions between the lipid matrix and fungal membrane components can overcome electrostatic repulsion, enabling effective drug association and cellular uptake.

### 5.5. Entrapment Efficiency (EE)

Entrapment efficiency (EE, %) describes the proportion of the drug or active ingredient successfully encapsulated within a carrier system relative to the total amount of drug used in the formulation. In contrast, drug loading (DL, %) refers to the mass ratio of the drug to the lipid carrier and determines the actual drug content within the nanosystem, which is particularly relevant for dosage considerations, excipient burden, and biocompatibility. Both parameters influence release behavior, bioavailability, and overall formulation performance.

High EE ensures that a large fraction of the added drug is retained within the nanocarrier, minimizing drug loss during production. High DL, on the other hand, enables the delivery of therapeutically relevant doses while reducing the amount of excipient required, which may improve biocompatibility. Increased payload and the lipidic nature of nanoparticles can contribute to prolonged and enhanced drug activity. Drug solubility in the lipid matrix is a key determinant of EE; for successful incorporation into SLNs or NLCs, the active molecule must possess sufficiently high solubility in the lipid selected for production [224].

Both EE and DL may be influenced by particle size and the physical state of the lipid matrix. However, while some studies report higher EE in smaller nanoparticles [225], this effect is highly dependent on lipid-drug interactions, the degree of crystallinity, and the preparation method, since reduced core volume may also limit the total drug payload. As discussed in Section 3, NLCs often show higher EE than SLNs due to their mixed solid–liquid lipid composition, which generates a less ordered crystalline structure capable of accommodating larger amounts of drug and reducing drug expulsion during storage. For example, ketoconazole-loaded SLNs and NLCs showed EE values up to 84.8% and 95.3%, respectively [221], while fluconazole-loaded SLNs and NLCs exhibited efficiencies of 75.7 ± 4.94% and 81.4 ± 3.89%, respectively [223]. Nevertheless, as discussed in Section 5.1, a high initial EE does not necessarily ensure long-term drug retention. Polymorphic transitions and lipid recrystallization during storage, particularly in SLNs, can promote drug expulsion and a progressive reduction in EE, highlighting the importance of considering lipid polymorphism and matrix stability alongside initial encapsulation values.

### 5.6. Drug Release Profile

Many LDDS formulations demonstrate sustained or prolonged drug release, reducing the risk of burst effects often observed with conventional drug solutions. Drug release profiles from lipid-based nanocarriers are commonly described using established mathematical models, such as the Higuchi model or the Korsmeyer–Peppas model. These models provide a standardized framework for interpreting release kinetics from LDDSs [226].

Many LDDSs display a characteristic biphasic release profile, consisting of an initial rapid release phase attributed to drug adsorbed or weakly bound at the particle surface, followed by a sustained release phase governed by diffusion from the lipid core. This pattern enables rapid attainment of therapeutic concentrations while maintaining prolonged drug exposure [227].

Drug release is influenced by several formulation-dependent factors, including the properties of the lipid matrix, drug solubility, particle size, and surfactant composition. Drug solubility and initial drug loading are key controlling factors for drug delivery. When the initial drug loading is below the solubility limit, release occurs primarily via simple diffusion through the lipid matrix. In contrast, when drug loading exceeds the solubility threshold, drug dissolution within the lipid becomes the rate-limiting factor [228]. NLCs generally exhibit faster release rates than SLNs [229,230], probably because the inclusion of liquid lipids in NLCs generates a less ordered, more imperfect crystalline matrix that offers fewer barriers to drug diffusion [231].

With respect to particle size, smaller particles exhibit a higher surface-to-volume ratio, which markedly increases the surface area available for drug release and enzymatic attack, thereby accelerating release kinetics [154]. In addition, the type of stabilizer used (e.g., Poloxamer 188 vs. lecithin) can either hinder or facilitate drug release by accelerating or decelerating lipid matrix degradation and influencing particle size evolution [132]. Sustained release is particularly beneficial for the treatment of chronic fungal infections or biofilm-associated diseases, where prolonged exposure to inhibitory drug concentrations is essential to prevent recurrence and resistance development [232].

## 6. Advantages and Disadvantages

Lipid-based drug delivery systems (LDDSs) provide multiple advantages for antifungal therapy by addressing limitations associated with conventional formulations and offering additional functionalities that improve drug performance, safety, and therapeutic efficacy [139,140].

A major advantage of LDDSs is their ability to enhance critical physicochemical and biopharmaceutical properties of antifungal agents in both experimental and clinical settings by overcoming intrinsic limitations such as low solubility, chemical instability, and poor bioavailability. As described in Section 4, lipid matrices efficiently encapsulate poorly water-soluble and chemically labile compounds [233,234]. In this context, the encapsulation of essential oils has gained increasing interest because of their broad antifungal activity—including efflux pump inhibition, a key mechanism underlying antimicrobial resistance—but their volatility and instability greatly limit clinical application [178,235]. The protection provided by LDDSs enables their therapeutic exploitation as antifungal agents and resistance modulators.

LDDSs can markedly reduce drug-related toxicity while preserving antifungal efficacy. This advantage is exemplified by lipid formulations of amphotericin B, which significantly decrease nephrotoxicity without compromising antifungal activity. The lipid environment stabilizes amphotericin B, limiting toxic self-aggregation and enhancing selectivity for fungal ergosterol over mammalian cholesterol, as discussed in detail in Section 4.2 [163,236]. Similar reductions in systemic or local toxicity have been achieved for other potent antifungals incorporated into lipid nanocarriers [237,238]. The improved safety profile of LDDSs is largely attributable to their intrinsic biocompatibility. These systems are composed of physiological, biodegradable, and biocompatible lipids, resulting in low acute and chronic toxicity and better tolerability than many non-lipid nanocarriers. Unlike inorganic nanocarriers (e.g., silica- or gold-based nanoparticles), LDDSs are fully biodegradable and do not accumulate in tissues, thereby avoiding long-term safety concerns associated with carrier persistence [122,132,154]. While other nanocarrier platforms also address formulation limitations, the combination of biocompatibility, safety, and translational feasibility represents a distinctive advantage of LDDSs. This translational potential is supported by relevant manufacturing advantages, as they can be produced using scalable and industry-compatible techniques, including high-pressure homogenization and microfluidics, often without toxic organic solvents. These approaches facilitate large-scale production and batch-to-batch reproducibility [172,221,239].

Regarding drug release, the solid or semi-solid lipid matrix of solid lipid nanoparticles (SLNs) and nanostructured lipid carriers (NLCs) intrinsically enables controlled and sustained release, maintaining therapeutic concentrations at the infection site over prolonged periods [240]. In contrast, conventional formulations often generate rapid release followed by subtherapeutic levels [213,241]. Sustained exposure improves patient adherence, reduces dosing frequency, and may help limit resistance development by avoiding fluctuating drug concentrations [138,159,168]. Although other lipid-based carriers, such as LPs or nanoemulsions, can also modulate drug release profiles depending on their formulation (including specialized or multilayered nanoemulsions), sustained and controlled release is a characteristic feature most consistently associated with SLNs and NLCs.

Another important advantage of LDDSs is their versatility across administration routes. For topical and mucosal delivery, their lipid composition mimics the stratum corneum, enhancing skin interaction and facilitating deeper penetration into skin, nails, or hair follicles—common reservoirs of fungal persistence [161,236]. Drug accumulation within hair follicles acts as a depot, supporting sustained local activity and reducing relapse [161]. In this context, NLCs, owing to their tailored lipid composition and nanometric size, have demonstrated enhanced follicular targeting compared to conventional ointments, enabling deeper penetration into hair follicles and improving the management of recalcitrant infections such as tinea capitis [242]. Beyond topical use, LDDSs have shown efficacy via ocular, oral, vaginal, and intravenous routes, promoting local drug accumulation while minimizing systemic exposure [132,243]. For oral administration, lipid carriers improve absorption through enhanced solubilization, lipid digestion-mediated uptake pathways, and lymphatic transport, thereby bypassing first-pass metabolism and increasing bioavailability [244,245]. This lymphatic uptake is particularly relevant for the treatment of systemic mycoses caused by pathogens such as *Cryptococcus* or *Histoplasma*, which disseminate via lymphatic and mononuclear phagocyte pathways during infection [246].

Importantly, LDDSs represent a promising strategy to overcome antifungal resistance. Encapsulation enhances intracellular drug accumulation, reduces the impact of efflux pumps, and promotes more efficient uptake by fungal cells [139,175,177]. Numerous studies report reduced MICs for antifungals loaded into SLNs or NLCs against *Candida* species, including resistant strains and emerging pathogens such as *C. auris* [148,150,247]. Enhanced antibiofilm activity and the possibility of co-delivering synergistic drug combinations further strengthen the resistance-modulating potential of these systems [161].

Despite these advantages, the clinical translation of LDDSs for antifungal therapy remains limited by several formulation, biological, manufacturing, and regulatory constraints.

One major formulation challenge is physicochemical instability, which can compromise drug loading and promote leakage during storage. These issues may affect all LDDS types [230,248], but are particularly well documented in SLNs due to the highly ordered nature of their crystalline lipid matrices, which tend to reorganize over time into more stable polymorphic forms with reduced space for drug accommodation [139,140,146,152,230]. This process may result in drug expulsion, especially for hydrophilic compounds, which generally exhibit low solubility in solid lipids and consequently low encapsulation efficiencies [132,247,249]. Although the incorporation of liquid lipids in NLCs disrupts crystallinity and improves stability, encapsulation of highly water-soluble drugs remains challenging [146,153]. Instability may also lead to particle aggregation, growth, or gelation during storage, increasing particle size and polydispersity, which may reduce their suitability for parenteral use [146]. Sterilization procedures required for injectable products may further promote particle enlargement or chemical degradation, compromising stability [140]. For thermolabile lipid systems, sterile filtration (0.22 µm) or aseptic processing are therefore often required as alternatives to heat sterilization; however, these approaches introduce additional challenges related to particle size constraints, process complexity, and manufacturing costs. Consequently, developing stable lipid formulations with high loading capacity is essential for maximizing the therapeutic potential of LDDS-based antifungal therapies.

From a therapeutic standpoint, the sustained release associated with SLNs and NLCs may paradoxically reduce apparent antifungal activity in vitro, leading to higher MICs or smaller inhibition zones compared with free drugs due to slower diffusion into culture media [158,168,169]. Strong drug-lipid interactions may further restrict release, reducing efficacy relative to free drug, as reported for miconazole- and econazole-loaded SLNs [250]. Conversely, uncontrolled burst release can lead to subtherapeutic concentrations over time, which is considered a potential driver of antifungal resistance [138]. In this context, time-kill kinetics assays may provide a more appropriate and accurate alternative to endpoint MIC determinations for LDDSs, as they better reflect their prolonged and time-dependent antifungal activity.

From a manufacturing perspective, although scalable methods exist, many techniques—including solvent evaporation, melt emulsification, and ultrasonic dispersion—pose challenges for industrial translation. They may require high temperatures, pressures, or solvent concentrations, which can degrade thermolabile drugs and complicate scale-up [251]. Achieving consistent particle size, drug loading, and stability requires stringent process control, while quality assessment is hindered by difficulties in distinguishing free from encapsulated drug, commonly addressed through techniques such as centrifugal ultrafiltration or dialysis [115,225,239]. High production costs, especially for established formulations such as liposomal amphotericin B, also limit accessibility, particularly in low-resource settings [150].

Finally, toxicological and regulatory concerns remain. Nanoscale systems—particularly those below 100 nm—may induce cytotoxic, nephrotoxic, hepatotoxic, or genotoxic effects due to their high surface-area-to-mass ratio. Surface charge density is also a critical determinant of biological interactions and toxicity, often influencing cellular uptake and membrane perturbation to an extent comparable to particle size [150]. The absence of harmonized regulatory frameworks for nanomedicines constitutes an additional barrier to clinical translation, emphasizing the need for standardized guidelines addressing safety, efficacy, scalability, and cost-effectiveness [115].

## 7. Application of LDDSs for Antifungal Purposes

As has already been discussed, application of LDDSs for the improvement of antifungal drug efficiency is a promising strategy to overcome antifungal resistance. Table 2 summarizes various antifungal-loaded LDDS formulations, their physicochemical properties, and the observed improvements in antifungal activity.

## 8. Future Perspectives

The rapid global rise in invasive fungal infections, their associated mortality rates exceeding 50%, and the limited availability of new antifungal agents underscore an urgent and unresolved clinical challenge, particularly in immunocompromised and critically ill patients. In this context, antifungal resistance not only compromises treatment outcomes but also leaves an increasingly restricted therapeutic arsenal/options. Against this backdrop, lipid-based drug delivery systems (LDDSs) are well positioned to play a central role in the future of antifungal therapy, particularly in addressing the accelerating challenge of antifungal resistance. Beyond their current role as solubilizing and stabilizing platforms, next-generation lipid nanocarriers are expected to function as multifunctional systems capable of overcoming resistance mechanisms, enabling precision targeting, and facilitating clinical translation.

A major future direction involves exploiting LDDSs to counteract resistance pathways. Strategies intended to enhance intracellular drug accumulation, shield antifungal agents from efflux pump recognition, and disrupt biofilm architecture are expected to become design principles in next-generation LDDSs. Early studies demonstrating azole resistance reversal in *Candida* spp. using fluconazole-loaded NLCs support the feasibility of this approach [176]. The encapsulation of poorly soluble antifungals (e.g., itraconazole) and bioactive natural compounds (e.g., essential oils with efflux-pump-inhibiting activity) further broadens the therapeutic repertoire against multidrug-resistant species, including *C. auris* [139,178,268].

Improving the efficacy and safety profile of existing antifungals remains another key priority. Next-generation LDDSs aim to further reduce the dose-limiting toxicities of agents such as amphotericin B while maintaining potency [162]. Enhancing the oral bioavailability of azoles, including ketoconazole and itraconazole, represents an important opportunity [160,221,268]. Co-delivery approaches, such as dual-drug LDDSs combining conventional antifungals with natural bioactive molecules, illustrate future trends toward synergistic, multi-mechanistic therapies [145,160,195,197].

Expanding advanced delivery routes and targeting strategies will shape the next phase of LDDS innovation. Advances include dermal and transungual systems designed for persistent superficial infections [161,269], ocular formulations capable of enhancing corneal penetration [165], and pulmonary-directed systems for invasive aspergillosis with improved pulmonary retention [172]. Emerging innovations such as pH-responsive nanoparticles, ligand-functionalized carriers with affinity for fungal cell wall components, and nanocarrier surface functionalization with cell-penetrating peptides are expected to further refine selective drug delivery [177,266,270]. In this context, advanced targeting strategies based on surface functionalization with specific ligands, antibodies, or aptamers directed against fungal cell wall components are expected to enhance selectivity and may contribute to the development of more personalized antifungal therapies [271]. In addition, enzyme-triggered release systems, designed to respond to fungal-secreted enzymes such as lipases or proteases, are emerging as a promising strategy to achieve more selective and site-specific drug release [272,273]. Furthermore, theranostic LDDSs, which combine antifungal drug delivery with diagnostic imaging agents, represent an emerging approach to enable real-time monitoring of drug distribution and therapeutic response, particularly in deep-seated fungal infections [274].

Finally, the translation of LDDSs to clinical practice will require overcoming technological and regulatory barriers. Recent studies emphasize the need for scalable, solvent-free manufacturing; improved long-term stability; and clear regulatory frameworks tailored to nanomedicines [115,150,239]. Progress in high-throughput screening, omics-based characterization and machine learning-guided formulation design may accelerate this transition by enabling the rational design of LDDSs that modulate fungal pathogenicity and resistance pathways [275].

In summary, LDDSs represent versatile and strategically positioned platforms to reshape antifungal therapy in the coming decade. Their clinical relevance lies in directly addressing pressing challenges such as rising antifungal resistance, the high mortality rates associated with invasive mycoses, and the limited availability of new antifungal agents. The future success of LDDS-based approaches will depend on optimizing stability, scalability, and safety, as well as on generating robust clinical evidence demonstrating meaningful improvements in patient outcomes. Ultimately, translating these technologies into routine clinical practice has the potential to alleviate the growing global burden of fungal disease and to expand the currently limited therapeutic arsenal available.

## Figures and Tables

**Table 1:** Comparison of structural and functional characteristics of LPs, SLNs, NLCs, and LNPs.

Feature	LPs	SLNs	NLCs	LNPs
Lipid Matrix	Phospholipids + cholesterol + PEG or targeting ligands	Solid lipids (solid at RT and body T°)	Solid + liquid lipids (imperfect matrix)	Phospholipids + ionizable lipids + cholesterol + PEG-lipids
Internal Structure	Vesicle-like	Highly ordered crystalline matrix	Less ordered, amorphous	Amorphous/non-crystalline, organized to incorporate nucleic acids
Main Cargo Type	Mostly hydrophilic but also lipophilic	Mostly lipophilic but also hydrophilic	Lipophilic and amphiphilic (higher loading)	Mainly nucleic acids (mRNA, siRNA, DNA)
Drug Loading Capacity	High (for hydrophobic drugs)	Moderate	High	Very high (for nucleic acids)
Release Profile	Faster release	Controlled, sustained	Controlled and stable	Rapid intracellular release (after endosomal escape)
Stability	Potential bilayer leakage	Risk of drug expulsion during storage	Improved stability (less crystallization)	High colloidal stability due to PEG-lipids
Particle Size	~50 nm–several µm	~50–300 nm	~50–300 nm	~60–150 nm
Key Advantages	Simple, safe, easily scalable	Simple, safe, stable, easily scalable	High drug loading, reduced leakage, flexible formulation	Efficient gene delivery, high transfection efficiency, scalable
Key Limitations	Low stability	Limited loading; crystallization issues	Require optimized lipid ratios	More complex formulation Higher production costs

LPs: liposomes; SLNs: solid lipid nanoparticles; NLCs: nanostructured lipid carriers; LNPs: lipid nanoparticles; RT: room temperature; T°: temperature; PEG: polyethylene glycol.

**Table 2:** Antifungal-loaded LDDS formulations: composition, physicochemical characteristics, and therapeutic improvements.

NP Type	Loaded Drug	Main Components	Size (nm)	PDI	ZP (mV)	EE %	Key Improvements	Ref.
NLC	Fluconazole	Compritol 888 ATO, Oleic acid, Lecithin, Poloxamer 188	~134.3	0.19	−29.0	81.4	5-fold higher drug accumulation in skin (rats) vs. the free drug	[223]
NLC	Itraconazole	Precirol ATO 5, Transcutol HP, Tween 80, Solutol HS15	313.7	0.562	−18.7	70.5	~2-fold increase in brain drug concentration (mice) vs. the free drug	[252]
NLC	Voriconazole (Gel)	Precirol ATO 5, Labrafil 1944 CS, Tween 80	193.5	0.272	−27.8	86.2	2.8-fold greater in vitro skin permeation and accumulation vs. conventional cream	[253]
NLC	Itraconazole	Precirol ATO 5, Oleic acid, Polysorbate 20	106	0.202	−28.7	99.9	Deep penetration into lungs and air sacs (falcon)	[173]
NLC	Ketoconazole	Dynasan 116, Castor oil, Soylecithin, Poloxamer 188	167.8	0.25	−32.5	95.3	2.7-fold enhancement in oral bioavailability (rats) vs. the free drug	[221]
NLC	Eugenol	Carnauba wax, Oleic acid, Poloxamer 407	199.5	0.07	−25.9	83.1	10- and 8-fold MIC reduction for *C. albicans* and *N. glabratus* vs. the free drug	[191]
NLC	Fluconazole	Stearic acid, Oleic acid, Tn80, Sn80	122.6	0.231	−36.7	95.3	Higher MIC reduction in azole-resistant *Candida* vs. fluconazole-SLN	[175]
NLC	*Lippia sidoides* Oil	Beeswax, Carnauba wax, oleic acid, SDS	307.8	0.22	−93.1	90–100	High activity against multidrug-resistant *C. auris*	[148]
SLN	Econazole	Tripalmitic glyceride, Stearylamine, Tween 80	18.7	0.25	+19.1	95.9	Best corneal permeability and ocular bioavailability (rabbits)	[165]
SLN	Griseofulvin	Stearic acid, Chitosan,	56.9	0.19	−34.8	94.8	2-fold improvement in bioavailability (rats) vs. the free drug	[152]
SLN	Clotrimazole	Softisan 100, Glyceryl oleate, Tween 80, DDAB	~100	<0.3	+35	96.7	Halved MIC values against 25 *C. albicans* strains vs. the free drug	[160]
SLN	Miconazole Nitrate	Gelucire 39/01, Glyceryl monostearate (GMS), Cremophor RH 40, Transcutol HP	244.2	0.22	−26.9	95.9	Ex vivo high skin drug retention (74.5%) vs. the free drug (52.6%)	[222]
SLN	AmB	Glyceryl palmitostearate, Poloxamer 407, Phospholipids	145.2	0.298	−15.3	93.2	2-fold reduction in MIC/MFC for *C. albicans* and *C. neoformans* vs. Fungizone^®^ and the free drug	[153]
SLN	Fluconazole	Compritol 888, PEG 600, Tween 80, Soya Lecithin	138.3	0.271	−2.1	62.1	165% higher flux through porcine cornea vs. Zocon^®^	[247]
SLN	Fluconazole	Compritol 888, Precirol ATO5, Poloxamer 407	292.0	0.228	−22.9	79	4-fold increase in eradication of pityriasis versicolor vs. Candistan^®^	[254]
SLN	Itraconazole	Stearic acid, PVA	139.1	0.078	−23.6	94.9	Higher ex vivo goat corneal permeability than palmitic-SLNs	[255]
SLN	Luliconazole	Gelucire 50/13, Precirol ATO 5, Tween 80	62.2	0.263	N/A	81.5	Sustained in vitro drug release (62% over 72 h) and high activity against *C. albicans*	[168]
SLN	Natamycin	Precirol ATO 5, Pluronic F68, stearyl amine	84.0	N/A	+26.0	~85	2.5-fold reduction in MIC against *A. fumigatus* and *C. albicans* vs. the free drug	[256]
SLN	Phytol	1,3-distearyl-2-oleyl-glycerol, PVA	302.2	0.12	−16.7	68.7	~300-fold reduction in MIC against *C. albicans*, *C. dubliniensis*, *C. parapsilosis*, and *N. glabratus* vs. the free drug	[158]
SLN	Sulconazole	GMS, Phospholipon 90H, Tween 20	89.8	0.311	−27	86.5	2.62-fold enhancement in skin permeation (rabbits) vs. the free drug	[159]
SLN	Terbinafine	GMS, Pluronic F68	241.3	0.415	−15.2	98.4	Higher in vitro and in vivo activity against *C. albicans* vs. Carbopol^®^	[147]
SLN	AmB	Stearic acid, Soy lecithin, Tween 80, Chitosan	158.9	0.13	+34.3	88.5	3.9- and 11.5-fold lower IC_50_ vs. AmBisome^®^ and Fungizone^®^, respectively	[257]
SLN	AmB	GMS, Soy lecithin, Tween 80	378.9	0.28	−35	97.8	~10-fold reduction in vitro hemolytic toxicity vs. the free drug	[163]
SLN	Butenafine HCl	Olivem 1000, Olivem 300, Stearyl amine	261.3	0.268	+24	91.4	Enhanced in vitro activity against *C. albicans* vs. the free drug	[258]
SLN	Clotrimazole	Precirol ATO 5, Polysorbate 80	450.6	N/A	−36	85	2-fold higher ex vivo skin permeation (rats) vs. the free drug	[174]
SLN	Itraconazole	Compritol 888 ATO, Tween 80, Stearylamine	262.92	N/A	+22.4	94.2	3.6-fold higher ex vivo skin retention (rats) and improved recovery from tinea pedis infection	[259]
SLN	Ketoconazole	Compritol 888 ATO, PEG 600, Phospholipon 90G	293	0.258	−22.9	88.5	410–900% increase in vivo skin retention (rats) vs. the free drug	[161]
SLN	Luliconazole	Glyceryl tristearate, Phospholipon 90H	290.7	0.494	−10.5	97.8	Higher ex vivo skin deposition in rats and mice	[236]
SLN	Terbinafine HCl	GMS, Tween 80, Thiourea	426.3	0.477	−24.1	89.8	Larger in vitro inhibition zone for *T. rubrum* (130 mm) vs. Lamifen^®^ (73 mm)	[260]
SLN	Oxiconazole Nitrate	Stearic acid, Poloxamer 407, Tween 80	101.4	0.31	−49	77.1	Superior clinical improvement in tinea infection vs. Tinox^®^	[261]
SLN	Terbinafine HCl	GMS, Tween 80, Plurol Oleique	148.6	0.305	−20.4	78	2-fold higher in vitro skin deposition and enhanced activity against *C. albicans* vs. the free drug	[262]
SLN	Griseofulvin (Gel)	Compritol 888 ATO, Tween 80, Phospholipon 90G	117	0.258	−11.0	66.4	5- and 3-fold higher skin permeation and retention, respectively, vs. conventional cream (rats)	[263]
Vesicular/Other	Voriconazole (LNP)	Compritol 888 ATO, DPPC, DMPG, Poloxamer 407	123.1	0.18	−16.2	83.9	3-fold reduction in *A. fumigatus* burden in BALF (rats)	[172]
Vesicular/Other	Luliconazole (LCNP)	Glyceryl monooleate, Poloxamer 407	181	0.26	+5.6	91.5	2-fold higher ex vivo transdermal flux and skin retention vs. marketed cream	[157]
Vesicular/Other	Anidulafungin (LP)	Hydrogenated soy phosphatidylcoline, phosphatidylglycerol, cholesterol	100	0.1	−74 to −79	94.6	Up to 99% reduction in fungal load in preformed *C. albicans* biofilm vs. the free drug	[192]
Vesicular/Other	Fluconazole (Cub)	Monoolein, 2-morpholinoethyl oleate, pluronic F-127	256	0.14	+28	59	Lower MIC_90_ values for *C. neoformans* vs. the free drug	[177]
Vesicular/Other	AmB (Supramol.)	Resorcinarene macrocycle, Cholesterol	279.4	0.18	−12.2	85.5	1.5-fold higher oral Cmax vs. free suspension (rabbits)	[245]
Vesicular/Other	Itraconazole (LNC)	Labrafac, Kolliphor HS 15, Lipoid S75	47.9	0.09	−15	99.8	Enhanced dermal retention in cutaneous candidiasis (rats)	[264]
Vesicular/Other	AmB and Thymus EO (Lipo-Niosome)	Tween 60, Cholesterol, DPPC, PEG	~200	0.32	−24.6	94.2	Reduced in vitro cytotoxicity of the co-encapsulated formulation (26%) vs. free combination (56%)	[265]
Vesicular/Other	Luliconazole and Niacinamide (Lipid nanocarrier)	Cholesterol, Soya-L-a-lecithin	126.4	0.276	−34.6	72.1	Higher in vivo activity against *C. albicans* vs. Candid V gel (rabbits)	[199]
Vesicular/Other	Voriconazole (Ethosomes)	Calcofluor white-PE conjugate, Phospholipids	~95	~0.24	−23.3	43.3	5- and 7.8-fold reduction in *C. albicans* burden in liver and kidney, respectively (mice)	[266]
Vesicular/Other	AmB (Cub)	Glyceryl monoolein, Poloxamer 407	192.3	0.20	Neg	94	5.1-fold increase in transport across Caco-2 cells vs. Fungizone^®^	[267]

AmB: amphotericin B; BALF: bronchoalveolar lavage fluid; Cub: cubosome; DDAB: dimethyldioctadecylammonium bromide; DMPG: dimyristoylphosphatodylglycerol; DPPC: dipalmitoylphosphatidylcholine; EE: entrapment efficiency; EO: essential oil; LCNP: liquid crystal nanoparticle; LNC: lipid nanocapsule; LP: liposome; N/A: not available; Neg: negative result but exact data not available; NLC: nanostructured lipid carrier; NP: nanoparticle; PDI: polydispersity index; PE: phosphatidylethanolamine; PEG: polyethylene glycol; PVA: polyvinyl alcohol; SDS: sodium dodecyl sulfate; SLN: solid lipid nanoparticle; ZP: zeta potential.

## Data Availability

No new data were created or analyzed in this study. Data sharing is not applicable to this article.

## Abbreviations

The following abbreviations are used in this manuscript:

aPDT	Antimicrobial photodynamic therapy
DDS	Drug Delivery System
DL	Drug Loading
EE	Entrapment Efficiency
EPS	Extracellular Polymeric Substance
GRAS	Generally Recognized as Safe
LDDS	Lipid-based Drug Delivery System
LNPs	Lipid Nanoparticles
LPs	Liposomes
MIC	Minimum Inhibitory Concentration
MFC	Minimum Fungicidal Concentration
NLCs	Nanostructured Lipid Carriers
SLNs	Solid Lipid Nanoparticles

## References

[1] Denning D.W. (2024). Global incidence and mortality of severe fungal disease. Lancet Infect. Dis..

[2] Bongomin F., Gago S., Oladele R.O., Denning D.W. (2017). Global and multi-national prevalence of fungal diseases—Estimate precision. J. Fungi.

[3] World Health Organization (2025). Antifungal Agents in Clinical and Preclinical Development Overview and Analysis.

[4] Salmanton-García J. (2024). Update on invasive fungal infections: Emerging trends in the incidence of fungal infections in immunosuppressed patients and associated conditions. Ther. Adv. Infect. Dis..

[5] Gras E., Azoyan L., Monzo-Gallo P., Garcia-Vidal C., Lanternier F., Brissot E., Guitard J., Lacombe K., Dechartres A., Surgers L. (2025). Risk factors for invasive mould infections in adult patients with hematological malignancies and/or stem cell transplant: A systematic literature review and meta-analysis. J. Infect..

[6] Zuniga-Moya J.C., Papadopoulos B., Mansoor A.-E.-R., Mazi P.B., Rauseo A.M., Spec A. (2024). Incidence and mortality of COVID-19-associated invasive fungal infections among critically ill intubated patients: A multicenter retrospective cohort analysis. Open Forum Infect. Dis..

[7] Nasir N., Kazmi S.A.M., Farooqi J., Irfan M., Jabeen K. (2025). Risk factors and clinical outcomes of invasive fungal infections in patients with severe COVID-19: A case–control study. Pathogens.

[8] Kriegl L., Egger M., Boyer J., Hoenigl M., Krause R. (2025). New treatment options for critically important WHO fungal priority pathogens. Clin. Microbiol. Infect..

[9] WHO (2022). Fungal Priority Pathogens List to Guide Research, Development and Public Health Action.

[10] Pfaller M.A., Diekema D.J., Turnidge J.D., Castanheira M., Jones R.N. (2019). Twenty years of the SENTRY antifungal surveillance program: Results for *Candida* species from 1997–2016. Open Forum Infectious Diseases.

[11] Hui S.T., Gifford H., Rhodes J. (2024). Emerging antifungal resistance in fungal pathogens. Curr. Clin. Microbiol. Rep..

[12] Lackner M., Alastruey-Izquierdo A., Armstrong-James D.P.H., Bromley M.J., Fisher M.C., Verweij P.E. (2025). Azole fungicides and Aspergillus resistance, five EU agency report highlights the problem for the first time using a One Health approach. npj Antimicrob. Resist..

[13] Lee Y., Robbins N., Cowen L.E. (2023). Molecular mechanisms governing antifungal drug resistance. npj Antimicrob. Resist..

[14] Kadariswantiningsih I.N., Empitu M.A., Santosa T.I., Alimu Y. (2025). Antifungal resistance: Emerging mechanisms and implications (Review). Mol. Med. Rep..

[15] Odds F.C., Brown A.J.P., Gow N.A.R. (2003). Antifungal agents: Mechanisms of action. Trends Microbiol..

[16] Shapiro R.S., Robbins N., Cowen L.E. (2011). Regulatory circuitry governing fungal development, drug resistance, and disease. Microbiol. Mol. Biol. Rev..

[17] Hargrove T.Y., Friggeri L., Wawrzak Z., Qi A., Hoekstra W.J., Schotzinger R.J., York J.D., Guengerich F.P., Lepesheva G.I. (2017). Structural analyses of *Candida albicans* sterol 14α-demethylase complexed with azole drugs address the molecular basis of azole-mediated inhibition of fungal sterol biosynthesis. J. Biol. Chem..

[18] Elsaman H., Golubtsov E., Brazil S., Ng N., Klugherz I., Martin R., Dichtl K., Müller C., Wagener J. (2024). Toxic eburicol accumulation drives the antifungal activity of azoles against *Aspergillus fumigatus*. Nat. Commun..

[19] Pappas P.G., Kauffman C.A., Andes D.R., Clancy C.J., Marr K.A., Ostrosky-Zeichner L., Reboli A.C., Schuster M.G., Vazquez J.A., Walsh T.J. (2016). Clinical practice guideline for the management of candidiasis: 2016 update by the infectious diseases society of america. Clin. Infect. Dis..

[20] Keighley C., Cooley L., Morris A.J., Ritchie D., Clark J.E., Boan P., Worth L.J., The Australasian Antifungal Guidelines Steering Committee (2021). Consensus guidelines for the diagnosis and management of invasive candidiasis in haematology, oncology and intensive care settings, 2021. Intern. Med. J..

[21] Cornely O.A., Sprute R., Bassetti M., Chen S.C.-A., Groll A.H., Kurzai O., Lass-Flörl C., Ostrosky-Zeichner L., Rautemaa-Richardson R., Revathi G. (2025). Global guideline for the diagnosis and management of candidiasis: An initiative of the ECMM in cooperation with ISHAM and ASM. Lancet Infect. Dis..

[22] Quindós G., Marcos-Arias C., Miranda-Cadena K., Sevillano E., Jauregizar N., Schneider J., Eraso E. (2025). The future of non-invasive azole antifungal treatment options for the management of vulvovaginal candidiasis. Expert Rev. Anti-Infect. Ther..

[23] Benitez L.L., Carver P.L. (2019). Adverse effects associated with long-term administration of azole antifungal agents. Drugs.

[24] Yang Y.-L., Xiang Z.-J., Yang J.-H., Wang W.-J., Xu Z.-C., Xiang R.-L. (2021). Adverse effects associated with currently commonly used antifungal agents: A network meta-analysis and systematic review. Front. Pharmacol..

[25] Santos A.L.S., Ramos L.S., Mello T.P., Viganor L., Nonato N.M.B.M., Pinheiro R.E.E., Branquinha M.H. (2025). Azole antifungals under pressure: Therapeutic challenges and multifaceted resistance mechanisms. Curr. Med. Chem..

[26] Sanglard D., Kuchler K., Ischer F., Pagani J.L., Monod M., Bille J. (1995). Mechanisms of resistance to azole antifungal agents in *Candida albicans* isolates from AIDS patients involve specific multidrug transporters. Antimicrob. Agents Chemother..

[27] Coste A., Turner V., Ischer F., Morschhäuser J., Forche A., Selmecki A., Berman J., Bille J., Sanglard D. (2006). A mutation in Tac1p, a transcription factor regulating CDR1 and CDR2, is coupled with loss of heterozygosity at chromosome 5 to mediate antifungal resistance in *Candida albicans*. Genetics.

[28] Schubert S., Barker K.S., Znaidi S., Schneider S., Dierolf F., Dunkel N., Aïd M., Boucher G., Rogers P.D., Raymond M. (2011). Regulation of efflux pump expression and drug resistance by the transcription factors Mrr1, Upc2, and Cap1 in *Candida albicans*. Antimicrob. Agents Chemother..

[29] Rybak J.M., Muñoz J.F., Barker K.S., Parker J.E., Esquivel B.D., Berkow E.L., Lockhart S.R., Gade L., Palmer G.E., White T.C. (2020). Mutations in TAC1B: A novel genetic determinant of clinical fluconazole resistance in *Candida* auris. mBio.

[30] Barker K.S., Santana D.J., Zhang Q., Peters T.L., Rybak J.M., Morschhäuser J., Cuomo C.A., Rogers P.D. (2025). Mutations in TAC1B drive increased CDR1 and MDR1 expression and azole resistance in *Candida auris*. Antimicrob. Agents Chemother..

[31] Esquivel B.D., Santos A., Rybak J.M., Santana D.J., Rogers P.D., White T.C. (2026). Mutations in ERG11, TAC1B, and CDR1 reduce fluconazole accumulation in drug-resistant *Candidozyma auris* isolates. mBio.

[32] Slaven J.W., Anderson M.J., Sanglard D., Dixon G.K., Bille J., Roberts I.S., Denning D.W. (2002). Increased expression of a novel *Aspergillus fumigatus* ABC transporter gene, atrF, in the presence of itraconazole in an itraconazole resistant clinical isolate. Fungal Genet. Biol..

[33] Marichal P., Koymans L., Willemsens S., Bellens D., Verhasselt P., Luyten W., Borgers M., Ramaekers F.C.S., Odds F.C., Vanden Bossche H. (1999). Contribution of mutations in the cytochrome P450 14α-demethylase (Erg11p, Cyp51p) to azole resistance in *Candida* albicans. Microbiology.

[34] Morio F., Loge C., Besse B., Hennequin C., Le Pape P. (2010). Screening for amino acid substitutions in the *Candida albicans* Erg11 protein of azole-susceptible and azole-resistant clinical isolates: New substitutions and a review of the literature. Diagn. Microbiol. Infect. Dis..

[35] Czajka K.M., Venkataraman K., Brabant-Kirwan D., Santi S.A., Verschoor C., Appanna V.D., Singh R., Saunders D.P., Tharmalingam S. (2023). Molecular mechanisms associated with antifungal resistance in pathogenic *Candida* species. Cells.

[36] Li J., Coste A.T., Liechti M., Bachmann D., Sanglard D., Lamoth F. (2021). Novel ERG11 and TAC1b mutations associated with azole resistance in *Candida auris*. Antimicrob. Agents Chemother..

[37] Sewell T.R., Zhu J., Rhodes J., Hagen F., Meis J.F., Fisher M.C., Jombart T. (2019). Nonrandom distribution of azole resistance across the global population of *Aspergillus fumigatus*. mBio.

[38] Rocchi S., Sewell T.R., Valot B., Godeau C., Laboissiere A., Millon L., Fisher M.C. (2021). Molecular epidemiology of azole-resistant *Aspergillus fumigatus* in france shows patient and healthcare links to environmentally occurring genotypes. Front. Cell. Infect. Microbiol..

[39] Dunkel N., Liu T.T., Barker K.S., Homayouni R., Morschhäuser J., Rogers P.D. (2008). A gain-of-function mutation in the transcription factor Upc2p causes upregulation of ergosterol biosynthesis genes and increased fluconazole resistance in a clinical *Candida albicans* isolate. Eukaryot. Cell.

[40] Li J., Aubry L., Brandalise D., Coste A.T., Sanglard D., Lamoth F. (2024). Upc2-mediated mechanisms of azole resistance in *Candida auris*. Microbiol. Spectr..

[41] Hagiwara D., Miura D., Shimizu K., Paul S., Ohba A., Gonoi T., Watanabe A., Kamei K., Shintani T., Moye-Rowley W.S. (2017). A novel Zn2-Cys6 transcription factor AtrR plays a key role in an azole resistance mechanism of *Aspergillus fumigatus* by co-regulating cyp51A and cdr1B expressions. PLoS Pathog..

[42] Du W., Zhai P., Wang T., Bromley M.J., Zhang Y., Lu L. (2021). The C2H2 transcription factor SltA contributes to azole resistance by coregulating the expression of the drug target Erg11A and the drug efflux pump Mdr1 in *Aspergillus fumigatus*. Antimicrob. Agents Chemother..

[43] Morio F., Pagniez F., Lacroix C., Miegeville M., Le Pape P. (2012). Amino acid substitutions in the *Candida albicans* sterol 5,6-desaturase (Erg3p) confer azole resistance: Characterization of two novel mutants with impaired virulence. J. Antimicrob. Chemother..

[44] Selmecki A., Forche A., Berman J. (2006). Aneuploidy and isochromosome formation in drug-resistant *Candida albicans*. Science.

[45] Santos R.S., Loureiro K.C., Rezende P.S., Andrade L.N., de Melo Barbosa R., Santini A., Santos A.C., Ferreira da Silva C., Souto E.B., de Sousa D.P. (2019). Innovative nanocompounds for cutaneous administration of classical antifungal drugs: A systematic review. J. Dermatol. Treat..

[46] Mroczyńska M., Brillowska-Dąbrowska A. (2020). Review on current status of echinocandins use. Antibiotics.

[47] Branco J., Miranda I.M., Rodrigues A.G. (2023). *Candida parapsilosis* virulence and antifungal resistance mechanisms: A comprehensive review of key determinants. J. Fungi.

[48] Aruanno M., Glampedakis E., Lamoth F. (2019). Echinocandins for the treatment of invasive aspergillosis: From laboratory to bedside. Antimicrob. Agents Chemother..

[49] Boyton T.T., Ferneini E.M. (2016). 9-Antimicrobial pharmacology for head, neck, and orofacial nonbacterial infections. Head, Neck, and Orofacial Infections.

[50] Perlin D.S. (2014). Echinocandin resistance, susceptibility testing and prophylaxis: Implications for patient management. Drugs.

[51] Howard K.C., Dennis E.K., Watt D.S., Garneau-Tsodikova S. (2020). A comprehensive overview of the medicinal chemistry of antifungal drugs: Perspectives and promise. Chem. Soc. Rev..

[52] Huang S.-J., Song Y.-H., Lv G., Liu J.-Y., Zhao J.-T., Wang L.-L., Xiang M.-J. (2025). Emergence of invasive candidiasis with multiple *Candida* species exhibiting azole and echinocandin resistance. Front. Microbiol..

[53] Gow N.A.R., Johnson C., Berman J., Coste A.T., Cuomo C.A., Perlin D.S., Bicanic T., Harrison T.S., Wiederhold N., Bromley M. (2022). The importance of antimicrobial resistance in medical mycology. Nat. Commun..

[54] WHO (2022). Guidelines for Diagnosing, Preventing and Managing Cryptococcal Disease Among Adults, Adolescents and Children Living with HIV.

[55] Garcia-Effron G., Katiyar S.K., Park S., Edlind T.D., Perlin D.S. (2008). A naturally occurring proline-to-alanine amino acid change in Fks1p in *Candida parapsilosis*, *Candida orthopsilosis*, and *Candida metapsilosis* accounts for reduced echinocandin susceptibility. Antimicrob. Agents Chemother..

[56] Kordalewska M., Lee A., Park S., Berrio I., Chowdhary A., Zhao Y., Perlin D.S. (2018). Understanding echinocandin resistance in the emerging pathogen *Candida auris*. Antimicrob. Agents Chemother..

[57] Barchiesi F., Spreghini E., Tomassetti S., Della Vittoria A., Arzeni D., Manso E., Scalise G. (2006). Effects of caspofungin against *Candida guilliermondii* and *Candida parapsilosis*. Antimicrob. Agents Chemother..

[58] Rocha E.M.F., Garcia-Effron G., Park S., Perlin D.S. (2007). A Ser678Pro substitution in Fks1p confers resistance to echinocandin drugs in *Aspergillus fumigatus*. Antimicrob. Agents Chemother..

[59] Andrés G.Q. (2015). Micología Clínica.

[60] Singh A., Singh J., Kumar S. (2025). Aspergillosis: A comprehensive review of pathogenesis, drug resistance, and emerging therapeutics. J. Food Drug Anal..

[61] Yang F., Teoh F., Tan A.S.M., Cao Y., Pavelka N., Berman J. (2019). Aneuploidy enables cross-adaptation to unrelated drugs. Mol. Biol. Evol..

[62] Yang F., Gritsenko V., Slor Futterman Y., Gao L., Zhen C., Lu H., Jiang Y.-Y., Berman J. (2021). Tunicamycin potentiates antifungal drug tolerance via aneuploidy in *Candida albicans*. mBio.

[63] Dickwella Widanage M.C., Gautam I., Sarkar D., Mentink-Vigier F., Vermaas J.V., Ding S.-Y., Lipton A.S., Fontaine T., Latgé J.-P., Wang P. (2024). Adaptative survival of *Aspergillus fumigatus* to echinocandins arises from cell wall remodeling beyond β-1,3-glucan synthesis inhibition. Nat. Commun..

[64] Cavassin F.B., Baú-Carneiro J.L., Vilas-Boas R.R., Queiroz-Telles F. (2021). Sixty years of amphotericin B: An overview of the main antifungal agent used to treat invasive fungal infections. Infect. Dis. Ther..

[65] Guo X., Zhang J., Li X., Xiao E., Lange J.D., Rienstra C.M., Burke M.D., Mitchell D.A. (2021). Sterol sponge mechanism is conserved for glycosylated polyene macrolides. ACS Cent. Sci..

[66] Ngece K., Ntondini T.L., Khwaza V., Paca A.M., Aderibigbe B.A. (2024). Polyene-based derivatives with antifungal activities. Pharmaceutics.

[67] Anderson T.M., Clay M.C., Cioffi A.G., Diaz K.A., Hisao G.S., Tuttle M.D., Nieuwkoop A.J., Comellas G., Maryum N., Wang S. (2014). Amphotericin forms an extramembranous and fungicidal sterol sponge. Nat. Chem. Biol..

[68] Carolus H., Pierson S., Lagrou K., Van Dijck P. (2020). Amphotericin B and other polyenes-discovery, clinical use, mode of action and drug resistance. J. Fungi.

[69] Quindós G., De-la-Pinta I., Marcos-Arias C., Jauregizar N., Sevillano E., Madariaga L., Eraso E. (2026). Therapeutic tools for vulvovaginal candidiasis: Current and emerging antifungal agents. J. Fungi.

[70] Shivarathri R., Jenull S., Chauhan M., Singh A., Mazumdar R., Chowdhary A., Kuchler K., Chauhan N. (2022). Comparative transcriptomics reveal possible mechanisms of amphotericin B resistance in *Candida auris*. Antimicrob. Agents Chemother..

[71] Chen M.-M., Shi G.-H., Dai Y., Fang W.-X., Wu Q. (2023). Identifying genetic variants associated with amphotericin B (AMB) resistance in *Aspergillus fumigatus* via k-mer-based GWAS. Front. Genet..

[72] Akinosoglou K., Rigopoulos E.A., Papageorgiou D., Schinas G., Polyzou E., Dimopoulou E., Gogos C., Dimopoulos G. (2024). Amphotericin B in the era of new antifungals: Where will it stand?. J. Fungi.

[73] Vermes A., Guchelaar H.J., Dankert J. (2000). Flucytosine: A review of its pharmacology, clinical indications, pharmacokinetics, toxicity and drug interactions. J. Antimicrob. Chemother..

[74] Sigera L.S.M., Denning D.W. (2023). Flucytosine and its clinical usage. Ther. Adv. Infect. Dis..

[75] Boyce K.J., Wang Y., Verma S., Shakya V.P.S., Xue C., Idnurm A. (2017). Mismatch repair of DNA replication errors contributes to microevolution in the pathogenic fungus *Cryptococcus neoformans*. mBio.

[76] Pfaller M.A., Messer S.A., Boyken L., Huynh H., Hollis R.J., Diekema D.J. (2002). In vitro activities of 5-fluorocytosine against 8,803 clinical isolates of *Candida* spp.: Global assessment of primary resistance using national committee for clinical laboratory standards susceptibility testing methods. Antimicrob. Agents Chemother..

[77] Delma F.Z., Al-Hatmi A.M.S., Brüggemann R.J.M., Melchers W.J.G., de Hoog S., Verweij P.E., Buil J.B. (2021). Molecular mechanisms of 5-fluorocytosine resistance in yeasts and filamentous fungi. J. Fungi.

[78] Abou-Chakra N., Astvad K.M.T., Martinussen J., Munksgaard A.S.E., Arendrup M.C. (2025). Exponential clonal expansion of 5-fluorocytosine–resistant *Candida tropicalis* and new insights into underlying molecular mechanisms. Emerg. Infect. Dis..

[79] Phan-Canh T., Nguyen-Le D.-M., Luu P.-L., Khunweeraphong N., Kuchler K. (2025). Rapid in vitro evolution of flucytosine resistance in *Candida auris*. mSphere.

[80] Hammoudi Halat D., Younes S., Mourad N., Rahal M. (2022). Allylamines, benzylamines, and fungal cell permeability: A review of mechanistic effects and usefulness against fungal pathogens. Membranes.

[81] Ryder N.S., Wagner S., Leitner I. (1998). In vitro activities of terbinafine against cutaneous isolates of *Candida albicans* and other pathogenic yeasts. Antimicrob. Agents Chemother..

[82] Caplan A.S., Gold J.A.W., Smith D.J., Ely J.W. (2014). Diagnosis and management of tinea infections. Am. Fam. Physician.

[83] Łabędź N., Navarrete-Dechent C., Kubisiak-Rzepczyk H., Bowszyc-Dmochowska M., Pogorzelska-Antkowiak A., Pietkiewicz P. (2023). Pityriasis versicolor—A narrative review on the diagnosis and management. Life.

[84] Vardanyan R., Hruby V. (2016). Chapter 33—Antifungal Drugs. Synthesis of Best-Seller Drugs.

[85] Shen J.J., Arendrup M.C., Verma S., Saunte D.M.L. (2021). The Emerging terbinafine-resistant *Trichophyton* epidemic: What is the role of antifungal susceptibility testing?. Dermatology.

[86] Gupta A.K., Susmita, Nguyen H.C., Liddy A., Economopoulos V., Wang T. (2025). Terbinafine resistance in *Trichophyton rubrum* and *Trichophyton indotineae*: A literature review. Antibiotics.

[87] Martinez-Rossi N.M., Peres N.T.A., Bitencourt T.A., Martins M.P., Rossi A. (2021). State-of-the-Art Dermatophyte Infections: Epidemiology Aspects, Pathophysiology, and Resistance Mechanisms. J. Fungi.

[88] Ishii M., Yamada T., Ishikawa K., Ichinose K., Monod M., Ohata S. (2021). The Ptk2-Pma1 pathway enhances tolerance to terbinafine in *Trichophyton rubrum*. Antimicrob. Agents Chemother..

[89] Gupta A.K., Wang T., Mann A., Piguet V., Chowdhary A., Bakotic W.L. (2025). Mechanisms of resistance against allylamine and azole antifungals in *Trichophyton*: A renewed call for innovative molecular diagnostics in susceptibility testing. PLoS Pathog..

[90] Yamada T., Maeda M., Alshahni M.M., Tanaka R., Yaguchi T., Bontems O., Salamin K., Fratti M., Monod M. (2017). Terbinafine resistance of *Trichophyton* clinical isolates caused by specific point mutations in the squalene epoxidase gene. Antimicrob. Agents Chemother..

[91] Martinez-Rossi N.M., Bitencourt T.A., Peres N.T.A., Lang E.A.S., Gomes E.V., Quaresemin N.R., Martins M.P., Lopes L., Rossi A. (2018). Dermatophyte resistance to antifungal drugs: Mechanisms and prospectus. Front. Microbiol..

[92] Lee A. (2021). Ibrexafungerp: First approval. Drugs.

[93] Pfaller M.A., Messer S.A., Motyl M.R., Jones R.N., Castanheira M. (2013). Activity of MK-3118, a new oral glucan synthase inhibitor, tested against *Candida* spp. by two international methods (CLSI and EUCAST). J. Antimicrob. Chemother..

[94] Berkow E.L., Angulo D., Lockhart S.R. (2017). In vitro activity of a novel glucan synthase inhibitor, SCY-078, against clinical isolates of *Candida auris*. Antimicrob. Agents Chemother..

[95] Quindós G., Miranda-Cadena K., San-Millán R., Borroto-Esoda K., Cantón E., Linares-Sicilia M.J., Hamprecht A., Montesinos I., Tortorano A.M., Prigitano A. (2022). In vitro antifungal activity of ibrexafungerp (SCY-078) against contemporary blood isolates from medically relevant species of *Candida*: A European study. Front. Cell. Infect. Microbiol..

[96] Rivero-Menendez O., Soto-Debran J.C., Cuenca-Estrella M., Alastruey-Izquierdo A. (2021). In vitro activity of ibrexafungerp against a collection of clinical isolates of *Aspergillus*, including cryptic species and Cyp51A mutants, using EUCAST and CLSI methodologies. J. Fungi.

[97] Gebremariam T., Alkhazraji S., Gu Y., Najvar L.K., Borroto-Esoda K., Patterson T.F., Filler S.G., Wiederhold N.P., Ibrahim A.S. (2024). Ibrexafungerp is efficacious in a neutropenic murine model of pulmonary mucormycosis as monotherapy and combined with liposomal amphotericin B. *Antimicrob*. Agents Chemother..

[98] Qiao J., Gao P., Jiang X., Fang H. (2013). In vitro antifungal activity of farnesyltransferase inhibitors against clinical isolates of *Aspergillus* and *Candida*. Ann. Clin. Microbiol. Antimicrob..

[99] Wang Y., Xu F., Nichols C.B., Shi Y., Hellinga H.W., Alspaugh J.A., Distefano M.D., Beese L.S. (2022). Structure-guided discovery of potent antifungals that prevent ras signaling by inhibiting protein farnesyltransferase. J. Med. Chem..

[100] Dai X., Liu X., Li J., Chen H., Yan C., Li Y., Liu H., Deng D., Wang X. (2024). Structural insights into the inhibition mechanism of fungal GWT1 by manogepix. Nat. Commun..

[101] Cha H., Won D., Kang S., Kim E.-S., Lee K.-A., Lee W.-J., Lee K.-T., Bahn Y.-S. (2025). The calcineurin pathway regulates extreme thermotolerance, cell membrane and wall integrity, antifungal resistance, and virulence in *Candida auris*. PLoS Pathog..

[102] Almajid A., Bazroon A., Al-Awami H.M., Albarbari H., Alqahtani I., Almutairi R., Alsuwayj A., Alahmadi F., Aljawad J., Alnimer R. (2024). Fosmanogepix: The novel anti-fungal agent’s comprehensive review of in vitro, in vivo, and current insights from advancing clinical trials. Cureus.

[103] Oliver J.D., Sibley G.E.M., Beckmann N., Dobb K.S., Slater M.J., McEntee L., du Pré S., Livermore J., Bromley M.J., Wiederhold N.P. (2016). F901318 represents a novel class of antifungal drug that inhibits dihydroorotate dehydrogenase. Proc. Natl. Acad. Sci. USA.

[104] Wiederhold N.P. (2020). Review of the novel investigational antifungal olorofim. J. Fungi.

[105] Wiederhold N.P., Law D., Birch M. (2017). Dihydroorotate dehydrogenase inhibitor F901318 has potent in vitro activity against *Scedosporium* species and *Lomentospora prolificans*. J. Antimicrob. Chemother..

[106] Wiederhold N.P., Najvar L.K., Jaramillo R., Olivo M., Birch M., Law D., Rex J.H., Catano G., Patterson T.F. (2018). The orotomide olorofim is efficacious in an experimental model of central nervous system coccidioidomycosis. Antimicrob. Agents Chemother..

[107] Jørgensen K.M., Astvad K.M.T., Hare R.K., Arendrup M.C. (2018). EUCAST determination of olorofim (F901318) susceptibility of mold species, method validation, and MICs. Antimicrob. Agents Chemother..

[108] Rivero-Menendez O., Cuenca-Estrella M., Alastruey-Izquierdo A. (2019). In vitro activity of olorofim (F901318) against clinical isolates of cryptic species of *Aspergillus* by EUCAST and CLSI methodologies. J. Antimicrob. Chemother..

[109] Yan Z., Hua H., Xu Y., Samaranayake L.P. (2012). Potent antifungal activity of pure compounds from traditional chinese medicine extracts against six oral *Candida* species and the synergy with fluconazole against azole-resistant *Candida albicans*. Evid. Based Complement. Altern. Med..

[110] Freires I.D.A., Murata R.M., Furletti V.F., Sartoratto A., de Alencar S.M., Figueira G.M., de Oliveira Rodrigues J.A., Duarte M.C.T., Rosalen P.L. (2014). *Coriandrum sativum* L. (Coriander) essential oil: Antifungal activity and mode of action on *Candida* spp., and molecular targets affected in human whole-genome expression. PLoS ONE.

[111] Massa N., Cantamessa S., Novello G., Ranzato E., Martinotti S., Pavan M., Rocchetti A., Berta G., Gamalero E., Bona E. (2018). Antifungal activity of essential oils against azole-resistant and azole-susceptible vaginal *Candida glabrata* strains. Can. J. Microbiol..

[112] Gil F., Laiolo J., Bayona-Pacheco B., Cannon R.D., Ferreira-Pereira A., Carpinella M.C. (2022). Extracts from Argentinian native plants reverse fluconazole resistance in *Candida* species by inhibiting the efflux transporters Mdr1 and Cdr1. BMC Complement. Med. Ther..

[113] Miranda-Cadena K., Dias M., Costa-Barbosa A., Collins T., Marcos-Arias C., Eraso E., Pais C., Quindós G., Sampaio P. (2021). Development and characterization of monoolein-based liposomes of carvacrol, cinnamaldehyde, citral, or thymol with anti-*Candida* activities. Antimicrob. Agents Chemother..

[114] Wainwright M., Maisch T., Nonell S., Plaetzer K., Almeida A., Tegos G.P., Hamblin M.R. (2017). Photoantimicrobials-are we afraid of the light?. Lancet Infect. Dis..

[115] Silva L.B.B.D., Castilho I.G., de Souza Silva F.A., Ghannoum M., Garcia M.T., Carmo P.H.F. (2025). Antimicrobial photodynamic therapy for superficial, skin, and mucosal fungal infections: An update. Microorganisms.

[116] Beirão S., Fernandes S., Coelho J., Faustino M.A.F., Tomé J.P.C., Neves M.G.P.M.S., Tomé A.C., Almeida A., Cunha A. (2014). Photodynamic inactivation of bacterial and yeast biofilms with a cationic porphyrin. Photochem. Photobiol..

[117] Diogo P., Fernandes C., Caramelo F., Mota M., Miranda I.M., Faustino M.a.F., Neves M.G.P.M.S., Uliana M.P., de Oliveira K.T., Santos J.M. (2017). Antimicrobial photodynamic therapy against endodontic enterococcus faecalis and *Candida albicans* mono and mixed biofilms in the presence of photosensitizers: A comparative study with classical endodontic irrigants. Front. Microbiol..

[118] Benoit D.S., Overby C.T., Sims K.R., Ackun-Farmmer M.A. (2020). Drug delivery systems. Biomaterials Science.

[119] Mishra V., Singh M., Mishra Y., Charbe N., Nayak P., Sudhakar K., Aljabali A.A.A., Shahcheraghi S.H., Bakshi H., Serrano-Aroca Á. (2021). Nanoarchitectures in management of fungal diseases: An overview. Appl. Sci..

[120] Müller R.H., Runge S., Ravelli V., Mehnert W., Thünemann A.F., Souto E.B. (2006). Oral bioavailability of cyclosporine: Solid lipid nanoparticles (SLN^®^) versus drug nanocrystals. Int. J. Pharm..

[121] Müller R.H., Shegokar R., Keck C.M. (2011). 20 years of lipid nanoparticles (SLN and NLC): Present state of development and industrial applications. Curr. Drug Discov. Technol..

[122] Sánchez-López E., Espina M., Doktorovova S., Souto E.B., García M.L. (2017). Lipid nanoparticles (SLN, NLC): Overcoming the anatomical and physiological barriers of the eye—Part II—Ocular drug-loaded lipid nanoparticles. Eur. J. Pharm. Biopharm..

[123] Bangham A.D., Horne R.W. (1964). Negative staining of phospholipids and their structural modification by surface-active agents as observed in the electron microscope. J. Mol. Biol..

[124] Guimarães D., Cavaco-Paulo A., Nogueira E. (2021). Design of liposomes as drug delivery system for therapeutic applications. Int. J. Pharm..

[125] Liu P., Chen G., Zhang J. (2022). A review of liposomes as a drug delivery system: Current status of approved products, regulatory environments, and future perspectives. Molecules.

[126] Hillery A.M. (1997). Supramolecular lipidic drug delivery systems: From laboratory to clinic A review of the recently introduced commercial liposomal and lipid-based formulations of amphotericin B. Adv. Drug Deliv. Rev..

[127] Hassanpour P., Hamishehkar H., Bahari Baroughi B., Baradaran B., Sandoghchian Shotorbani S., Mohammadi M., Shomali N., Aghebati-Maleki L., Nami S. (2021). Antifungal effects of voriconazole-loaded nano-liposome on fluconazole-resistant clinical isolates of *Candida albicans*, biological activity and ERG11, CDR1, and CDR2 gene expression. Assay. Drug Dev. Technol..

[128] Müller R.H., Mäder K., Gohla S. (2000). Solid lipid nanoparticles (SLN) for controlled drug delivery—A review of the state of the art. Eur. J. Pharm. Biopharm..

[129] Arana L., Gallego L., Alkorta I. (2021). Incorporation of antibiotics into solid lipid nanoparticles: A promising approach to reduce antibiotic resistance emergence. Nanomaterials.

[130] Mu H., Holm R. (2018). Solid lipid nanocarriers in drug delivery: Characterization and design. Expert Opin. Drug Deliv..

[131] AlQurashi D.M., AlQurashi T.F., Alam R.I., Shaikh S., Tarkistani M.A.M. (2025). Advanced nanoparticles in combating antibiotic resistance: Current innovations and future directions. J. Nanotheranostics.

[132] Alvarez-Trabado J., Diebold Y., Sanchez A. (2017). Designing lipid nanoparticles for topical ocular drug delivery. Int. J. Pharm..

[133] Souto E.B., Fangueiro J.F., Fernandes A.R., Cano A., Sanchez-Lopez E., Garcia M.L., Severino P., Paganelli M.O., Chaud M.V., Silva A.M. (2022). Physicochemical and biopharmaceutical aspects influencing skin permeation and role of SLN and NLC for skin drug delivery. Heliyon.

[134] Udepurkar A., Devos C., Sagmeister P., Destro F., Inguva P., Ahmadi S., Boulais E., Quan Y., Braatz R.D., Myerson A.S. (2025). Structure and morphology of lipid nanoparticles for nucleic acid drug delivery: A review. ACS Nano.

[135] Paroor S., Shaji A.T., Bino L., Praveen N.G., Unnipurath S., Anil P., Geo R., Nagamalleswari E., Bonam S.R., Kurapati R. (2025). Lipid nanoparticles for the delivery of mRNA. Methods Mol. Biol..

[136] Li X., Li J., Wei J., Du W., Su C., Shen X., Zhao A., Xu M. (2025). Design strategies for novel lipid nanoparticle for mRNA vaccine and therapeutics: Current understandings and future perspectives. MedComm.

[137] Mundhe A.K., Rajkumari R. (2025). Overcoming antifungal resistance in *Candida albicans* via RNA interference: A therapeutic perspective. Front. Cell. Infect. Microbiol..

[138] Garg A., Sharma G.S., Goyal A.K., Ghosh G., Si S.C., Rath G. (2020). Recent advances in topical carriers of anti-fungal agents. Heliyon.

[139] Araujo V.H.S., Filippo L.D.D., Duarte J.L., Spósito L., de Camargo B.A.F., da Silva P.B., Chorilli M. (2021). Exploiting solid lipid nanoparticles and nanostructured lipid carriers for drug delivery against cutaneous fungal infections. Crit. Rev. Microbiol..

[140] Almawash S. (2023). Solid lipid nanoparticles, an effective carrier for classical antifungal drugs. Saudi Pharm. J..

[141] Prajapati S.K., Jain A., Bajpai M. (2025). Lipid-based nanoformulations in onychomycosis therapy: Addressing challenges of current therapies and advancing treatment. RSC Adv..

[142] Holmes A.R., Cardno T.S., Strouse J.J., Ivnitski-Steele I., Keniya M.V., Lackovic K., Monk B.C., Sklar L.A., Cannon R.D. (2016). Targeting efflux pumps to overcome antifungal drug resistance. Future Med. Chem..

[143] Dos Santos Ramos M.A., Da Silva P.B., Spósito L., De Toledo L.G., Bonifácio B.V., Rodero C.F., Dos Santos K.C., Chorilli M., Bauab T.M. (2018). Nanotechnology-based drug delivery systems for control of microbial biofilms: A review. Int. J. Nanomed..

[144] Carbone C., Teixeira M.D.C., Sousa M.d.C., Martins-Gomes C., Silva A.M., Souto E.M.B., Musumeci T. (2019). Clotrimazole-loaded mediterranean essential oils NLC: A synergic treatment of *Candida* skin infections. Pharmaceutics.

[145] Negi P., Singh A., Pundir S., Parashar A., Upadhyay N., Agarwal S., Chauhan R., Tambuwala M.M. (2023). Essential oil and nanocarrier-based formulations approaches for vaginal candidiasis. Ther. Deliv..

[146] Soliman G.M. (2017). Nanoparticles as safe and effective delivery systems of antifungal agents: Achievements and challenges. Int. J. Pharm..

[147] Rarokar N.R., Menghani S.S., Kerzare D.R., Khedekar P.B., Bharne A.P., Alamri A.S., Alsanie W.F., Alhomrani M., Sreeharsha N., Asdaq S.M.B. (2022). Preparation of terbinafin-encapsulated solid lipid nanoparticles containing antifungal Carbopol^®^ hydrogel with improved efficacy: In vitro, ex vivo and in vivo study. Pharmaceutics.

[148] Baldim I., Paziani M.H., Grizante Barião P.H., Kress M.R.V.Z., Oliveira W.P. (2022). Nanostructured lipid carriers loaded with *Lippia sidoides* essential oil as a strategy to combat the multidrug-resistant *Candida auris*. Pharmaceutics.

[149] Folle C., Marqués A.M., Díaz-Garrido N., Carvajal-Vidal P., Sánchez López E., Suñer-Carbó J., Halbaut L., Mallandrich M., Espina M., Badia J. (2024). Gel-dispersed nanostructured lipid carriers loading thymol designed for dermal pathologies. Int. J. Nanomed..

[150] Marena G.D., Ruiz-Gaitán A., Bauab T.M., Chorilli M. (2025). Improving antifungal lipid-based drug delivery against *Candida*: A review. Expert Opin. Drug Deliv..

[151] Saeidi Z., Giti R., Emami A., Rostami M., Mohammadi F. (2024). Thermosensitive and mucoadhesive gels containing solid lipid nanoparticles loaded with fluconazole and niosomes loaded with clindamycin for the treatment of periodontal diseases: A laboratory experiment. BMC Oral Health.

[152] Sreeharsha N., Prasanthi S., Rao G.S.N.K., Gajula L.R., Biradar N., Goudanavar P., Naveen N.R., Shiroorkar P.N., Meravanige G., Telsang M. (2025). Formulation optimization of chitosan surface coated solid lipid nanoparticles of griseofulvin: A Box-Behnken design and in vivo pharmacokinetic study. Eur. J. Pharm. Sci..

[153] Jansook P., Pichayakorn W., Ritthidej G.C. (2018). Amphotericin B-loaded solid lipid nanoparticles (SLNs) and nanostructured lipid carrier (NLCs): Effect of drug loading and biopharmaceutical characterizations. Drug Dev. Ind. Pharm..

[154] Dudhipala N., Ali Youssef A.A., Banala N. (2020). Colloidal lipid nanodispersion enriched hydrogel of antifungal agent for management of fungal infections: Comparative in-vitro, ex-vivo and in-vivo evaluation for oral and topical application. Chem. Phys. Lipids.

[155] Thanki K., Date T., Jain S. (2023). Enabling oral amphotericin B delivery by merging the benefits of prodrug approach and nanocarrier-mediated drug delivery. ACS Biomater. Sci. Eng..

[156] Guada M., Lasa-Saracíbar B., Lana H., Dios-Viéitez M.D.C., Blanco-Prieto M.J. (2016). Lipid nanoparticles enhance the absorption of cyclosporine A through the gastrointestinal barrier: In vitro and in vivo studies. Int. J. Pharm..

[157] Mahmood A., Rapalli V.K., Waghule T., Gorantla S., Singhvi G. (2021). Luliconazole loaded lyotropic liquid crystalline nanoparticles for topical delivery: QbD driven optimization, in-vitro characterization and dermatokinetic assessment. Chem. Phys. Lipids.

[158] Lima T.L.C., Souza L.B.F.C., Tavares-Pessoa L.C.S., dos Santos-Silva A.M., Cavalcante R.S., de Araújo-Júnior R.F., Cornélio A.M., Fernandes-Pedrosa M.F., Chaves G.M., da Silva-Júnior A.A. (2020). Phytol-loaded solid lipid nanoparticles as a novel anticandidal nanobiotechnological approach. Pharmaceutics.

[159] Samee A., Usman F., Wani T.A., Farooq M., Shah H.S., Javed I., Ahmad H., Khan R., Zargar S., Kausar S. (2023). Sulconazole-loaded solid lipid nanoparticles for enhanced antifungal activity: In vitro and in vivo approach. Molecules.

[160] Carbone C., Fuochi V., Zielińska A., Musumeci T., Souto E.B., Bonaccorso A., Puglia C., Petronio Petronio G., Furneri P.M. (2020). Dual-drugs delivery in solid lipid nanoparticles for the treatment of *Candida albicans* mycosis. Colloids Surf. B Biointerfaces.

[161] Ramzan M., Gourion-Arsiquaud S., Hussain A., Gulati J.S., Zhang Q., Trehan S., Puri V., Michniak-Kohn B., Kaur I.P. (2022). In vitro release, ex vivo penetration, and in vivo dermatokinetics of ketoconazole-loaded solid lipid nanoparticles for topical delivery. Drug Deliv. Transl. Res..

[162] Das S., Devarajan P.V. (2020). Enhancing safety and efficacy by altering the toxic aggregated state of amphotericin B in lipidic nanoformulations. Mol. Pharm..

[163] Joyson N., Pathak A., Jain K. (2023). One platform comparison of polymeric and lipidic nanoparticles for the delivery of amphotericin B. AAPS PharmSciTech.

[164] Chaudhari M.B., Desai P.P., Patel P.A., Patravale V.B. (2016). Solid lipid nanoparticles of amphotericin B (AmbiOnp): In vitro and in vivo assessment towards safe and effective oral treatment module. Drug Deliv. Transl. Res..

[165] Liang Z., Zhang Z., Lu P., Yang J., Han L., Liu S., Zhou T., Li J., Zhang J. (2023). The effect of charges on the corneal penetration of solid lipid nanoparticles loaded Econazole after topical administration in rabbits. Eur. J. Pharm. Sci..

[166] Kaur I.P., Kakkar S. (2010). Topical delivery of antifungal agents. Expert Opin. Drug Deliv..

[167] Trombino S., Servidio C., Laganà A.S., Conforti F., Marrelli M., Cassano R. (2020). Viscosified solid lipidic nanoparticles based on naringenin and linolenic acid for the release of cyclosporine A on the skin. Molecules.

[168] Firdaus S., Hassan N., Mirza M.A., Ara T., El-Serehy H.A., Al-Misned F.A., Iqbal Z. (2021). FbD directed fabrication and investigation of luliconazole based SLN gel for the amelioration of candidal vulvovaginitis: A 2 T (thermosensitive & transvaginal) approach. Saudi J. Biol. Sci..

[169] Hirun N., Mahadlek J., Limmatvapirat S., Sriamornsak P., Yonemochi E., Furuishi T., Kraisit P. (2024). Fabrication and characterization of pectin films containing solid lipid nanoparticles for buccal delivery of fluconazole. Int. J. Mol. Sci..

[170] Kakkar S., Karuppayil S.M., Raut J.S., Giansanti F., Papucci L., Schiavone N., Kaur I.P. (2015). Lipid-polyethylene glycol based nano-ocular formulation of ketoconazole. Int. J. Pharm..

[171] Aljurbui S.J., Hussain A., Yusuf M., Ramzan M., Afzal O., Almohaywi B., Yasmin S., Altamimi A.S.A. (2022). Impact of composition and morphology of ketoconazole-loaded solid lipid nanoparticles on intestinal permeation and gastroplus-based prediction studies. ACS Omega.

[172] Fayyaz H.A., EL-Massik M.A., Bahey-El-Din M., Abdel-Bary A., Abdallah O.Y., Eltaher H.M. (2024). Targeted DPPC/DMPG surface-modified voriconazole lipid nanoparticles control invasive pulmonary aspergillosis in immunocompromised population: In-vitro and in-vivo assessment. Int. J. Pharm..

[173] Pardeike J., Weber S., Zarfl H.P., Pagitz M., Zimmer A. (2016). Itraconazole-loaded nanostructured lipid carriers (NLC) for pulmonary treatment of aspergillosis in falcons. Eur. J. Pharm. Biopharm..

[174] Patel V., Mehta T., Shah J., Soni K. (2025). Quality by design driven development of lipid nanoparticles for cutaneous targeting: A preliminary approach. Drug Deliv. Transl. Res..

[175] Moazeni M., Saeedi M., Kelidari H., Nabili M., Davari A. (2019). An update on the application of nano-scaled carriers against fluconazole-resistant *Candida* species: Nanostructured lipid carriers or solid lipid nanoparticles?. Curr. Med. Mycol..

[176] Moazeni M., Kelidari H.R., Saeedi M., Morteza-Semnani K., Nabili M., Gohar A.A., Akbari J., Lotfali E., Nokhodchi A. (2016). Time to overcome fluconazole resistant *Candida* isolates: Solid lipid nanoparticles as a novel antifungal drug delivery system. Colloids Surf. B Biointerfaces.

[177] Rajesh S., Gangadoo S., Nguyen H., Zhai J., Dekiwadia C., Drummond C.J., Chapman J., Truong V.K., Tran N. (2022). Application of fluconazole-loaded pH-sensitive lipid nanoparticles for enhanced antifungal therapy. ACS Appl. Mater. Interfaces.

[178] Fuentes J.M., Jofré I., Tortella G., Benavides-Mendoza A., Diez M.C., Rubilar O., Fincheira P. (2024). The mechanistic insights of essential oil of Mentha piperita to control Botrytis cinerea and the prospection of lipid nanoparticles to its application. Microbiol. Res..

[179] da Silva E.F., dos Santos F.A.L., Pires H.M., Bastos L.M., Ribeiro L.N.d.M. (2025). Lipid nanoparticles carrying essential oils for multiple applications as antimicrobials. Pharmaceutics.

[180] Venishetty V.K., Komuravelli R., Kuncha M., Sistla R., Diwan P.V. (2013). Increased brain uptake of docetaxel and ketoconazole loaded folate-grafted solid lipid nanoparticles. Nanomed. Nanotechnol. Biol. Med..

[181] Garg A., Singh S. (2011). Enhancement in antifungal activity of eugenol in immunosuppressed rats through lipid nanocarriers. Colloids Surf. B Biointerfaces.

[182] Parvez S., Yadagiri G., Karole A., Singh O.P., Verma A., Sundar S., Mudavath S.L. (2020). Recuperating biopharmaceutical aspects of amphotericin B and paromomycin using a chitosan functionalized nanocarrier via oral route for enhanced anti-leishmanial activity. Front. Cell. Infect. Microbiol..

[183] Gilbert A.S., Wheeler R.T., May R.C. (2015). Fungal pathogens: Survival and replication within macrophages. Cold Spring Harb. Perspect. Med..

[184] Parvez S., Yadagiri G., Arora K., Javaid A., Kushwaha A.K., Singh O.P., Sundar S., Mudavath S.L. (2023). Coalition of biological agent (melatonin) with chemotherapeutic agent (amphotericin B) for combating visceral leishmaniasis via oral administration of modified solid lipid nanoparticles. ACS Biomater. Sci. Eng..

[185] Kulkarni J.A., Chen S., Tam Y.Y.C. (2021). Scalable production of lipid nanoparticles containing amphotericin B. Langmuir.

[186] Vyas S.P., Khatri K., Goyal A.K. (2009). Functionalized nanocarrier(s) to image and target fungi infected immune cells. Med. Mycol..

[187] Kuhn D.M., George T., Chandra J., Mukherjee P.K., Ghannoum M.A. (2002). Antifungal susceptibility of *Candida* biofilms: Unique efficacy of amphotericin B lipid formulations and echinocandins. Antimicrob. Agents Chemother..

[188] Jøraholmen M.W., Vanić Ž., Tho I., Škalko-Basnet N. (2014). Chitosan-coated liposomes for topical vaginal therapy: Assuring localized drug effect. Int. J. Pharm..

[189] Elhabal S.F., Faheem A.M., Hababeh S., Nelson J., Elzohairy N.A., Ibrahim Y.F., Ewedah T.M., Mousa I.S., Allam K.M., Hamdan A.M.E. (2025). Augmented marshmallow extract lipid nanoparticles with clove oil embedded in collagen sponge for ultimate antimicrobial healing of diabetic mouth ulcer. Pharmaceutics.

[190] de Alteriis E., Maione A., Falanga A., Bellavita R., Galdiero S., Albarano L., Salvatore M.M., Galdiero E., Guida M. (2021). Activity of free and liposome-encapsulated essential oil from lavandula angustifolia against persister-derived biofilm of *Candida auris*. Antibiotics.

[191] Guedes I.L., do Nascimento M.O., Dias L.D.S., de Araujo-Nobre A.R., Barreto H.M., Abi-Chacra É.d.A., Fialho A.C.V., Vale G.C., Carvalho A.L.M. (2025). Lipid nanocarrier containing eugenol for denture hygiene: Evaluation of efficacy against *Candida* biofilms. J. Appl. Oral Sci..

[192] Vera-González N., Bailey-Hytholt C.M., Langlois L., de Camargo Ribeiro F., de Souza Santos E.L., Junqueira J.C., Shukla A. (2020). Anidulafungin liposome nanoparticles exhibit antifungal activity against planktonic and biofilm *Candida albicans*. J. Biomed. Mater. Res. Part A.

[193] Marena G.D., Ramos M.A.D.S., Carvalho G.C., de Lima L.C., do Nascimento A.L.C.S., Sábio R.M., Rodero C.F., Spósito L., Bauab T.M., Chorilli M. (2022). Development and characterization of an amphotericin B—Loaded nanoemulsion applied to *Candida auris* biofilms control. J. Drug Deliv. Sci. Technol..

[194] Sato M.R., Oshiro-Junior J.A., Rodero C.F., Boni F.I., Araújo V.H.S., Bauab T.M., Nicholas D., Callan J.F., Chorilli M. (2023). Enhancing antifungal treatment of *Candida albicans* with hypericin-loaded nanostructured lipid carriers in hydrogels: Characterization, in vitro, and in vivo photodynamic evaluation. Pharmaceuticals.

[195] Ibrahim Al-Samadi I.E., Omar Rashwan K., Abdelmonem R., Hamed M.I.A., Darwish K.M., Magdy William M., Abdellatif M.M. (2025). Topical loratadine-loaded invasomal gel repurposed for vulvovaginal candidiasis; in vitro, in silico, ex vivo, and in vivo studies. Int. J. Pharm..

[196] Marena G.D., Carvalho G.C., Ruiz-Gaitán A., Onisto G.S., Bugalho B.C.M., Genezini L.M.V., Santos M.O.D., Blanco A.L., Chorilli M., Bauab T.M. (2024). Potential activity of micafungin and amphotericin B co-encapsulated in nanoemulsion against systemic *Candida auris* infection in a mice model. J. Fungi.

[197] Bezerra C.F., De Alencar Júnior J.G., De Lima Honorato R., Dos Santos A.T.L., Pereira Da Silva J.C., Gusmão Da Silva T., Leal A.L.A.B., Rocha J.E., De Freitas T.S., Tavares Vieira T.A. (2020). Antifungal activity of farnesol incorporated in liposomes and associated with fluconazole. Chem. Phys. Lipids.

[198] Bezerra C.F., Júnior J.G.D.A., Honorato R.d.L., dos Santos A.T.L., da Silva J.C.P., da Silva T.G., de Freitas T.S., Vieira T.A.T., Bezerra M.C.F., Lima Sales D. (2021). Antifungal effect of liposomal α-bisabolol and when associated with fluconazole. Cosmetics.

[199] Satapathy B.S., Zafar A., Warsi M.H., Behera S., Mohanty D.I., Mujtaba M.A., Mohanty M., Upadhyay A.K., Khalid M. (2025). Luliconazole–niacinamide lipid nanocarrier laden gel for enhanced treatment of vaginal candidiasis: In vitro, ex vivo, in silico and preclinical insights. RSC Adv..

[200] Gamirov R., Akhmedov A., Burdyugov D., Panina Y., Bukharov M., Sokolova E., Subakaeva E., Bukarinova Y., Zelenikhin P., Shurpik D. (2025). Combined antifungal nanocarriers based on self-assembled nystatin and pillar[5]arene with a terpene moiety. Org. Biomol. Chem..

[201] Nogueira N.C., de Sá L.L.F., de Carvalho A.L.M. (2021). Nanostructured lipid carriers as a novel strategy for topical antifungal therapy. AAPS PharmSciTech.

[202] Puglia C., Esposito E., Menegatti E., Nastruzzi C., Rizza L., Cortesi R., Bonina F. (2005). Effect of charge and lipid concentration on in-vivo percutaneous absorption of methyl nicotinate from liposomal vesicles. J. Pharm. Pharmacol..

[203] Kobierski J., Wnętrzak A., Chachaj-Brekiesz A., Dynarowicz-Latka P. (2022). Predicting the packing parameter for lipids in monolayers with the use of molecular dynamics. Colloids Surf. B Biointerfaces.

[204] Poojari C., Zak A., Dzieciuch-Rojek M., Bunker A., Kepczynski M., Róg T. (2020). Cholesterol reduces partitioning of antifungal drug itraconazole into lipid bilayers. J. Phys. Chem. B.

[205] Uner M., Wissing S.A., Yener G., Müller R.H. (2004). Influence of surfactants on the physical stability of solid lipid nanoparticle (SLN) formulations. Pharmazie.

[206] Danaei M., Dehghankhold M., Ataei S., Hasanzadeh Davarani F., Javanmard R., Dokhani A., Khorasani S., Mozafari M.R. (2018). Impact of particle size and polydispersity index on the clinical applications of lipidic nanocarrier systems. Pharmaceutics.

[207] Hoshyar N., Gray S., Han H., Bao G. (2016). The effect of nanoparticle size on in vivo pharmacokinetics and cellular interaction. Nanomedicine.

[208] Zeb A., Qureshi O.S., Kim H.-S., Cha J.-H., Kim H.-S., Kim J.-K. (2016). Improved skin permeation of methotrexate via nanosized ultradeformable liposomes. Int. J. Nanomed..

[209] Jenning V., Gysler A., Schäfer-Korting M., Gohla S.H. (2000). Vitamin A loaded solid lipid nanoparticles for topical use: Occlusive properties and drug targeting to the upper skin. Eur. J. Pharm. Biopharm..

[210] Ahmed S., Amin M.M., Sayed S. (2023). Ocular drug delivery: A comprehensive review. AAPS PharmSciTech.

[211] Wang L., Hu C., Shao L. (2017). The antimicrobial activity of nanoparticles: Present situation and prospects for the future. Int. J. Nanomed..

[212] Farkas N., Kramar J.A. (2021). Dynamic Light Scattering Distributions by Any Means. J. Nanopart Res..

[213] Jain S., Jain S., Khare P., Gulbake A., Bansal D., Jain S.K. (2010). Design and development of solid lipid nanoparticles for topical delivery of an anti-fungal agent. Drug Deliv..

[214] Ainurofiq A., Suryanto A.A., Beltiartono B.S., Merdekawati N.A., Ardiyani N.P., Farohma Q.Y.C., Budiman A., Wardhana Y.W., Nugraha Y.P. (2025). Literature review: The role of particle size distribution in drug delivery. Multidiscip. Rev..

[215] Alnusaire T.S. (2021). Olive leaves (*Olea Europaea* L) extract loaded lipid nanoparticles: Optimization of processing parameters by box-behnken statistical design, in-vitro characterization, and evaluation of anti-oxidant and anti-microbial activity. J. Oleo Sci..

[216] Németh Z., Csóka I., Semnani Jazani R., Sipos B., Haspel H., Kozma G., Kónya Z., Dobó D.G. (2022). Quality by design-driven zeta potential optimisation study of liposomes with charge imparting membrane additives. Pharmaceutics.

[217] Tamboli A.M.M., Tade J.M. (2025). Zeta Potential: A Comprehensive Review. Int. Res. J. Pharm. Med. Sci. IRJPMS.

[218] Irimia T., Ghica M.V., Popa L., Anuţa V., Arsene A.-L., Dinu-Pîrvu C.-E. (2018). Strategies for improving ocular drug bioavailability and corneal wound healing with chitosan-based delivery systems. Polymers.

[219] Kaminski K., Skora M., Krzyściak P., Stączek S., Zdybicka-Barabas A., Cytryńska M. (2021). Synthesis and study of antifungal properties of new cationic beta-glucan derivatives. Pharmaceuticals.

[220] Vieira D.B., Carmona-Ribeiro A.M. (2006). Cationic lipids and surfactants as antifungal agents: Mode of action. J. Antimicrob. Chemother..

[221] Dudhipala N., Ay A.A. (2020). Amelioration of ketoconazole in lipid nanoparticles for enhanced antifungal activity and bioavailability through oral administration for management of fungal infections. Chem. Phys. Lipids.

[222] Al-Maghrabi P.M., Khafagy E.-S., Ghorab M.M., Gad S. (2020). Influence of formulation variables on miconazole nitrate-loaded lipid based nanocarrier for topical delivery. Colloids Surf. B Biointerfaces.

[223] Gupta M., Vyas S.P. (2012). Development, characterization and in vivo assessment of effective lipidic nanoparticles for dermal delivery of fluconazole against cutaneous candidiasis. Chem. Phys. Lipids.

[224] Dudhipala N., Janga K.Y., Gorre T. (2018). Comparative study of nisoldipine-loaded nanostructured lipid carriers and solid lipid nanoparticles for oral delivery: Preparation, characterization, permeation and pharmacokinetic evaluation. Artif. Cells Nanomed. Biotechnol..

[225] Kupetz E., Bunjes H. (2014). Lipid nanoparticles: Drug localization is substance-specific and achievable load depends on the size and physical state of the particles. J. Control. Release.

[226] Porbaha P., Ansari R., Kiafar M.R., Bashiry R., Khazaei M.M., Dadbakhsh A., Azadi A. (2024). A comparative mathematical analysis of drug release from lipid-based nanoparticles. AAPS PharmSciTech.

[227] Wu K.-W., Sweeney C., Dudhipala N., Lakhani P., Chaurasiya N.D., Tekwani B.L., Majumdar S. (2021). Primaquine loaded solid lipid nanoparticles (SLN), nanostructured lipid carriers (NLC), and nanoemulsion (NE): Effect of lipid matrix and surfactant on drug entrapment, in vitro release, and ex vivo hemolysis. AAPS PharmSciTech.

[228] Vaghasiya H., Kumar A., Sawant K. (2013). Development of solid lipid nanoparticles based controlled release system for topical delivery of terbinafine hydrochloride. Eur. J. Pharm. Sci..

[229] Souto E.B., Wissing S.A., Barbosa C.M., Müller R.H. (2004). Development of a controlled release formulation based on SLN and NLC for topical clotrimazole delivery. Int. J. Pharm..

[230] Das S., Ng W.K., Tan R.B.H. (2012). Are nanostructured lipid carriers (NLCs) better than solid lipid nanoparticles (SLNs): Development, characterizations and comparative evaluations of clotrimazole-loaded SLNs and NLCs?. Eur. J. Pharm. Sci..

[231] Kim J.-K., Park J.-S., Kim C.-K. (2010). Development of a binary lipid nanoparticles formulation of itraconazole for parenteral administration and controlled release. Int. J. Pharm..

[232] Mba I.E., Nweze E.I. (2021). Nanoparticles as therapeutic options for treating multidrug-resistant bacteria: Research progress, challenges, and prospects. World J. Microbiol. Biotechnol..

[233] Wang T., Luo Y. (2018). Chitosan hydrogel beads functionalized with thymol-loaded solid lipid–polymer hybrid nanoparticles. Int. J. Mol. Sci..

[234] Talarico L., Clemente I., Gennari A., Gabbricci G., Pepi S., Leone G., Bonechi C., Rossi C., Mattioli S.L., Detta N. (2024). Physiochemical characterization of lipidic nanoformulations encapsulating the antifungal drug natamycin. Nanomaterials.

[235] Fincheira P., Espinoza J., Levío-Raimán M., Vera J., Tortella G., Brito A.M.M., Seabra A.B., Diez M.C., Quiroz A., Rubilar O. (2024). Formulation of essential oils-loaded solid lipid nanoparticles-based chitosan/PVA hydrogels to control the growth of *Botrytis cinerea* and *Penicillium expansum*. Int. J. Biol. Macromol..

[236] Kaur M., Singh G., Shivgotra R., Singh M., Thakur S., Jain S.K. (2024). Prolonged skin retention of luliconazole from SLNs based topical gel formulation contributing to ameliorated antifungal activity. AAPS PharmSciTech.

[237] Khan A.S., Ud Din F., Ali Z., Bibi M., Zahid F., Zeb A., Khan G.M. (2021). Development, in vitro and in vivo evaluation of miltefosine loaded nanostructured lipid carriers for the treatment of Cutaneous Leishmaniasis. Int. J. Pharm..

[238] Jacobus Berlitz S., Reginatto P., Machado G.D.R.M., Fuentefria A.M., Morisso F.D.P., Contri R.V., Külkamp-Guerreiro I.C. (2023). Development of a clioquinol nanocarrier as a new, promising option for the treatment of dermatomycosis. Pharmaceutics.

[239] Terada T., Kanou M., Hashimoto Y., Tanimoto M., Sugimoto M. (2022). Microfluidic preparation of nanoparticles using poly(ethylene glycol)-distearoylphosphatidylethanolamine for solubilizing poorly soluble drugs. J. Pharm. Sci..

[240] Zeb A., Qureshi O.S., Kim H.-S., Kim M.-S., Kang J.-H., Park J.-S., Kim J.-K. (2017). High payload itraconazole-incorporated lipid nanoparticles with modulated release property for oral and parenteral administration. J. Pharm. Pharmacol..

[241] Kaur I.P., Rana C., Singh H. (2008). Development of effective ocular preparations of antifungal agents. J. Ocul. Pharmacol. Ther..

[242] Heydari S., Barzegar-Jalali M., Heydari M., Radmehr A., Paiva-Santos A.C., Kouhsoltani M., Hamishehkar H. (2024). The impact of particle size of nanostructured lipid carriers on follicular drug delivery: A comprehensive analysis of mouse and human hair follicle penetration. Bioimpacts.

[243] Aljaeid B.M., Hosny K.M. (2016). Miconazole-loaded solid lipid nanoparticles: Formulation and evaluation of a novel formula with high bioavailability and antifungal activity. Int. J. Nanomed..

[244] Wang K., Qi J., Weng T., Tian Z., Lu Y., Hu K., Yin Z., Wu W. (2014). Enhancement of oral bioavailability of cyclosporine A: Comparison of various nanoscale drug-delivery systems. Int. J. Nanomed..

[245] Ali I., Burki S., ur Rehman J., Ullah S., Javid I., Abdellattif M.H., Shah M.R. (2023). Synthetic star shaped tetra-tailed biocompatible supramolecular amphiphile as an efficient nanocarrier for Amphotericin B. Chem. Phys. Lipids.

[246] Bednarek J.M., Brown J.C.S. (2024). Elements of dissemination in cryptococcosis. mBio.

[247] Kakkar S., Singh M., Mohan Karuppayil S., Raut J.S., Giansanti F., Papucci L., Schiavone N., Nag T.C., Gao N., Yu F.-S.X. (2021). Lipo-PEG nano-ocular formulation successfully encapsulates hydrophilic fluconazole and traverses corneal and non-corneal path to reach posterior eye segment. J. Drug Target..

[248] Tenchov R., Bird R., Curtze A.E., Zhou Q. (2021). Lipid nanoparticles—From liposomes to mRNA vaccine delivery, a landscape of research diversity and advancement. ACS Nano.

[249] Amekyeh H., Billa N. (2021). Lyophilized drug-loaded solid lipid nanoparticles formulated with beeswax and theobroma oil. Molecules.

[250] Shah R.M., Eldridge D.S., Palombo E.A., Harding I.H. (2017). Microwave-assisted microemulsion technique for production of miconazole nitrate- and econazole nitrate-loaded solid lipid nanoparticles. Eur. J. Pharm. Biopharm..

[251] Butani D., Yewale C., Misra A. (2016). Topical Amphotericin B solid lipid nanoparticles: Design and development. Colloids Surf. B Biointerfaces.

[252] Lim W.M., Rajinikanth P.S., Mallikarjun C., Kang Y.B. (2014). Formulation and delivery of itraconazole to the brain using a nanolipid carrier system. Int. J. Nanomed..

[253] Song S.H., Lee K.M., Kang J.B., Lee S.G., Kang M.J., Choi Y.W. (2014). Improved skin delivery of voriconazole with a nanostructured lipid carrier-based hydrogel formulation. Chem. Pharm. Bull..

[254] El-Housiny S., Shams Eldeen M.A., El-Attar Y.A., Salem H.A., Attia D., Bendas E.R., El-Nabarawi M.A. (2017). Fluconazole-loaded solid lipid nanoparticles topical gel for treatment of pityriasis versicolor: Formulation and clinical study. Drug Deliv..

[255] Mohanty B., Majumdar D.K., Mishra S.K., Panda A.K., Patnaik S. (2015). Development and characterization of itraconazole-loaded solid lipid nanoparticles for ocular delivery. Pharm. Dev. Technol..

[256] Khames A., Khaleel M.A., El-Badawy M.F., El-Nezhawy A.O.H. (2019). Natamycin solid lipid nanoparticles—Sustained ocular delivery system of higher corneal penetration against deep fungal keratitis: Preparation and optimization. Int. J. Nanomed..

[257] Jain V., Gupta A., Pawar V.K., Asthana S., Jaiswal A.K., Dube A., Chourasia M.K. (2014). Chitosan-assisted immunotherapy for intervention of experimental leishmaniasis via amphotericin B-loaded solid lipid nanoparticles. Appl. Biochem. Biotechnol..

[258] Baviskar A., Kashid V., Ahirrao S., Bhambere D., Akul M. (2024). Formulation and evaluation of butenafine hydrochloride-incorporated solid lipid nanoparticles as novel excipients for the treatment of superficial fungal infections. Turk. J. Pharm. Sci..

[259] Kumar N., Goindi S. (2021). Development and optimization of itraconazole-loaded solid lipid nanoparticles for topical administration using high shear homogenization process by design of experiments: In vitro, ex vivo and in vivo evaluation. AAPS PharmSciTech.

[260] Abobakr F.E., Fayez S.M., Elwazzan V.S., Sakran W. (2021). Effect of different nail penetration enhancers in solid lipid nanoparticles containing terbinafine hydrochloride for treatment of onychomycosis. AAPS PharmSciTech.

[261] Mahmoud R.A., Hussein A.K., Nasef G.A., Mansour H.F. (2020). Oxiconazole nitrate solid lipid nanoparticles: Formulation, in-vitro characterization and clinical assessment of an analogous loaded carbopol gel. Drug Dev. Ind. Pharm..

[262] Wavikar P., Vavia P. (2013). Nanolipidgel for enhanced skin deposition and improved antifungal activity. AAPS PharmSciTech.

[263] Aggarwal N., Goindi S. (2013). Preparation and in vivo evaluation of solid lipid nanoparticles of griseofulvin for dermal use. J. Biomed. Nanotechnol..

[264] El-Sheridy N.A., Ramadan A.A., Eid A.A., El-Khordagui L.K. (2019). Itraconazole lipid nanocapsules gel for dermatological applications: In vitro characteristics and treatment of induced cutaneous candidiasis. Colloids Surf. B Biointerfaces.

[265] Rahimi F., Amoabediny G., Sabahi H., Zandieh-Doulabi B. (2022). Fungal infected adipose stem cells: The effects of novel lipo-niosome nanoparticles loaded with amphotericin B and thymus essential oil. Cell J..

[266] Shen T., Li M., Tian B., Liu W., Chu L., Yu P., Zhou H., Han Y., Ding C., Sai S. (2024). Calcofluor white-phosphatidylethanolamine conjugate-enhanced ethosomal delivery of voriconazole for targeting *Candida albicans*. Int. J. Nanomed..

[267] Yang Z., Chen M., Yang M., Chen J., Fang W., Xu P. (2014). Evaluating the potential of cubosomal nanoparticles for oral delivery of amphotericin B in treating fungal infection. Int. J. Nanomed..

[268] Passos J.S., Martino L.C.D., Dartora V.F.C., Araujo G.L.B.D., Ishida K., Lopes L.B. (2020). Development, skin targeting and antifungal efficacy of topical lipid nanoparticles containing itraconazole. Eur. J. Pharm. Sci..

[269] Krawczyk-Santos A.P., Da Rocha P.B.R., Kloppel L.L., Souza B.D.S., Anjos J.L.V., Alonso A., De Faria D.L.A., Gil O.M., Gratieri T., Marreto R.N. (2021). Enhanced nail delivery of voriconazole-loaded nanomicelles by thioglycolic acid pretreatment: A study of protein dynamics and disulfide bond rupture. Int. J. Pharm..

[270] Khakshur A.A., Khodaverdi E., Kamali H., Nokhodchi A. (2025). An insight into cell-penetrating peptides: Perspectives on design, optimization, and targeting in drug delivery systems. Pharm. Dev. Technol..

[271] Wu S., Song R., Liu T., Li C. (2023). Antifungal therapy: Novel drug delivery strategies driven by new targets. Adv. Drug Deliv. Rev..

[272] Uroro E.O., Bright R., Hayles A., Vasilev K. (2022). Lipase-responsive amphotericin B loaded PCL nanoparticles for antifungal therapies. Nanomaterials.

[273] Vera-González N., Deusenbery C., LaMastro V., Shukla A. (2024). Fungal enzyme-responsive hydrogel drug delivery platform for triggered antifungal release. Adv. Healthc. Mater..

[274] Liu H., Zhong W., Zhang X., Lin D., Wu J. (2021). Nanomedicine as a promising strategy for the theranostics of infectious diseases. J. Mater. Chem. B.

[275] Mulat M., Banicod R.J.S., Tabassum N., Javaid A., Karthikeyan A., Jeong G.-J., Kim Y.-M., Jung W.-K., Khan F. (2025). Multiple strategies for the application of medicinal plant-derived bioactive compounds in controlling microbial biofilm and virulence properties. Antibiotics.

